# Knowledge and Technology Used in Capacitive Deionization of Water

**DOI:** 10.3390/membranes12050459

**Published:** 2022-04-24

**Authors:** Kamran Salari, Payam Zarafshan, Morteza Khashehchi, Gholamreza Chegini, Hamed Etezadi, Hamed Karami, Joanna Szulżyk-Cieplak, Grzegorz Łagód

**Affiliations:** 1 Department of Agrotechnology, Aburaihan Campus, University of Tehran, Tehran 14155-6619, Iran; m.khashehchi@ut.ac.ir (M.K.); chegini@ut.ac.ir (G.C.); hetezadi@ut.ac.ir (H.E.); 2 Department of Biosystems Engineering, University of Mohaghegh Ardabili, Ardabil 56199-11367, Iran; hamedkarami@uma.ac.ir; 3 Faculty of Technology Fundamentals, Lublin University of Technology, 20-618 Lublin, Poland; j.szulzyk-cieplak@pollub.pl; 4 Faculty of Environmental Engineering, Lublin University of Technology, 20-618 Lublin, Poland; g.lagod@pollub.pl

**Keywords:** desalination, capacitive deionization, Membrane Capacitive Deionization, cathode and anode permeable membrane, activated carbon, Electrical Double Layers, porous media

## Abstract

The demand for water and energy in today’s developing world is enormous and has become the key to the progress of societies. Many methods have been developed to desalinate water, but energy and environmental constraints have slowed or stopped the growth of many. Capacitive Deionization (CDI) is a very new method that uses porous carbon electrodes with significant potential for low energy desalination. This process is known as deionization by applying a very low voltage of 1.2 volts and removing charged ions and molecules. Using capacitive principles in this method, the absorption phenomenon is facilitated, which is known as capacitive deionization. In the capacitive deionization method, unlike other methods in which water is separated from salt, in this technology, salt, which is a smaller part of this compound, is separated from water and salt solution, which in turn causes less energy consumption. With the advancement of science and the introduction of new porous materials, the use of this method of deionization has increased greatly. Due to the limitations of other methods of desalination, this method has been very popular among researchers and the water desalination industry and needs more scientific research to become more commercial.

## 1. Introduction

**Figure 1:**
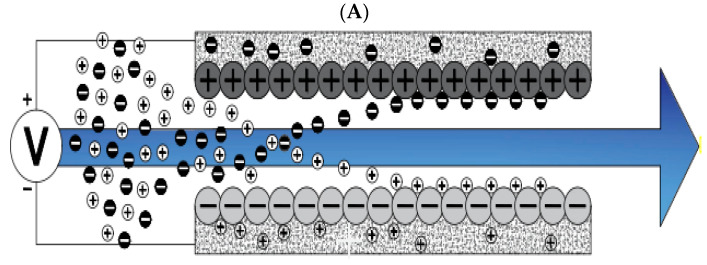

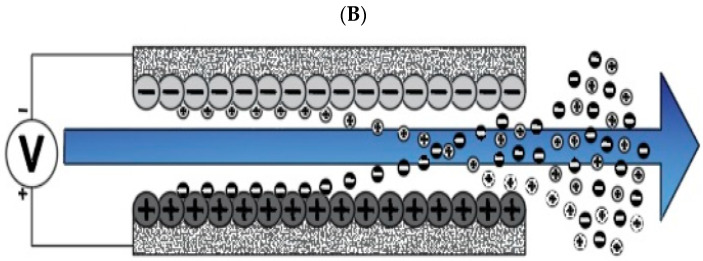
Water desalination process with capacitive deionization method. (**A**) The phase of ion adsorption (**B**), Electrode Regeneration steps.

Now that we are in the 21st century, despite the great developments in recent decades, we still face a fundamental problem called water scarcity. Water scarcity occurs because of the high consumption of water in different applications when available water resources cannot meet the demand. According to the FAO (Food and Agriculture Organization), more than 1.8 billion people in the world in 2025 will face severe water shortages [1,2]. The world’s fresh water is being used for domestic, industrial, and agricultural uses. According to the World Bank report, the annual consumption of fresh water resources is in three sectors: agriculture, industry, and domestic, with the largest share in the agricultural sector at 70% (this number reaches 92% in Iran), followed by industry at 20%, and the rest is related to domestic use [3]. Encountering water scarcity is one of the new human needs that leads to the adaptation of methods and techniques to deal with the issue. These methods include better management of available fresh water, recovery of wasted water, and desalination of available saline water sources [4,5]. Complicating matters with increasing groundwater extraction around the world lead to increased salinity in wells and aquifers. On the other hand, in many technological processes used in various branches of industry, including mining, significant amounts of wastewater with a high degree of mineralization are generated [6]. The reuse of such water by industry contributes to the protection of water resources and also helps to comply with the strict regulations regarding wastewater disposal. Therefore, there is a strong and attractive desire to develop desalination technologies economically. The main methods of desalination in the world are of three forms: thermal process, membrane process, and electrical (chemical) method, the choice of which is based on the criteria including the economics of desalination, salinity, quality of water produced, supply and access to energy and fuel with reasonable price, system simplicity and ease of use and device size, and low construction and maintenance costs [7,8,9,10]. The common goal for ongoing research and the emergence of various methods is that these technologies attempt an economical way to desalinate seawater and brackish water [11]. The limiting and important factor in choosing the type of desalination water is the amount of energy consumed and the amount of access to it so that 85 to 90% of the total energy in the desalination process is spent on deionization. Another important issue that has limited the water in desalination plants is their environmental effects. Today, research is being conducted to reduce chemical excretion, noise pollution, greenhouse gases, etc. [4,12,13]. Therefore, choosing a desalination process that has the least energy consumption and the least environmental impact is very important [13,14]. Capacitive deionization has emerged in recent years as a new and powerful technology in terms of energy efficiency and cost-effectiveness for low or medium salt desalination. It also introduces a new generation of desalination techniques without producing any harmful chemicals and without any membranes [15,16]. The efficiency of the capacitive method is that instead of removing the larger amount from the mixture of water and salt, it separates the smaller part of this mixture, which is salt, which is unlike other methods that separate water from salt. In addition, the release of energy during the electrode regeneration phase (discharge of the electrode) can be used as the energy required for another cell of the CDI unite, which is in the electrode charge phase, thereby recovering energy is becoming possible. As will be explained in more detail later, a CDI cycle consists of two stages, the first being ion adsorption or charging, known as the water purification step, in which ions are placed in pairs of porous electrodes. In the next step, the adsorbed ions are released, this means they are expelled from the electrodes, and thus the electrodes are regenerated. The mechanism of the capacitive deionization process is schematically shown in Figure 1.

With increasing research on porous carbon electrodes over the past decades for use in energy storage devices, the capacitive deionization method has been somewhat neglected. The use of porous carbon electrodes for desalination plants began in the 1960s, and research institutes are quickly testing and using this technology in the desalination industry. CDI or capacitive deionization uses a pair of oppositely placed porous carbon electrodes that store and absorb ions by applying a potential difference (Figure 1). These dual electrodes can be assembled in different batches and introduced as a single cell. The ions are absorbed by a mixture of water and salt flowing between the two electrodes and settling in the pores of the carbon material of the electrode. The formation of Electrical Double Layers (EDL) is the basis of capacitive energy storage as well as the mechanism by which immobile salt ions are selectively extracted from saline water. After a while, the entire volume in the pores of the carbon electrode particles is saturated with charged ions of salt and reaches the storage capacity of the electrodes. To regenerate saturated carbon electrodes, ions are released by reducing or even reversing the cell voltage from the electrode, producing a small ion-enriched current and the electrodes regain their original ion absorption capacity. This process can be considered completely physical without considering any chemical reaction, which makes CDI systems have a longer life and less maintenance. These dual electrodes can be assembled in different batches and introduced as a single cell. The ions are absorbed by a mixture of water and salt flowing between the two electrodes and settle in the pores of the carbon material of the electrode. The formation of Electrical Double Layers (EDL) is the basis of capacitive energy storage as well as the mechanism by which immobile salt ions are selectively extracted from saline water. After a while, the entire volume in the pores of the carbon electrode particles is saturated with charged ions of salt and reaches the storage capacity of the electrodes. Various modifications and new technologies have now been defined for CDI systems, including the exposure of ion thin membrane exchange in front of electrodes (MCDI (Membrane Capacitive Deionization)) [17], studying operating modes such as stop-flow operations during ion diffusion [18], salt release at reversed voltage [19], the constant current function [20], energy recovery from the desalination/release cycle [8,21], flow through the electrodes where the water insert from above the electrodes [22], and flow-electrodes based on carbon suspensions [23]. According to the materials used to fabricate the electrodes, a very basic question about the best material remains unanswered, and design strategies for new and improved electrodes are still ongoing [24]. The selection of electrode materials is largely dependent on the required performance (desalination capacity, final salt concentration), system requirements and design (flow rate and CDI cell structure), and financial issues (service and maintenance costs and durability).

This article is a review of the principles and theory of capacitive deionization of water and the history and materials used to make carbon electrodes, as well as the performance criteria of this method, have been reviewed and presented.

## 2. Background of Research on Capacitive Deionization Method

In this section, we will review the research that has been carried out to develop CDI since its inception. Initially, the research focused on the theoretical issues of this method, and after the scientific understanding of this issue, more research was conducted on the materials used to make the electrodes and researchers are still focused on the commercialization of this method and its application in various fields. Although some papers have noted the introduction of this method to be in 1957 by the University of Oklahoma [25]. The first research on the concept of capacitive desalination was conducted in 1960 by Blair and Murphy entitled Electrochemical Demineralization of Water which lasted until the end of the 1960s [26]. During that period, electrodes were selected based on ionic representations as selective anodes and cathodes (such as the cathode and anode permeable membrane) and it was hypothesized that ions could be separated from water when a specific chemical group was present on their surface after the ionic bonding of ions in the solution and the ionized groups on the carbon surface endured oxidation or reduction. The electrode material used in these experiments was graphite or other forms of carbon [27]. In 1961, Arnold and Murphy focused on developing a method for distinguishing between the selective nature of anions and cations for the electrode material, leading to the preparation of an anion selection electrode with the help of biomaterials [28]. In 1966, Evans and Hamilton studied the electrochemical demineralization of water by measuring the electric charge based on coulometric and mass balance. This study was performed on the development of ion absorption in the absence of an external potential difference [29]. In 1967, Murphy and Caudle presented the first detailed study of the deionization process with analytical relationships based on capacitive performance. This study, which was defined as the basis of the demineralization process, was discussed by combining the equilibrium equations of mass and ion transport and the dependence of the amount of TDS and EC on time, and the model was used subjected to several different operating conditions and numerous laboratory data [30]. In 1968, a study was conducted by Reid et al. to investigate the removal of polyvalent ions. In this study, it was observed that the demineralization system can be economically effective not only for the adsorption of sodium and chlorine ions (salt), which are monovalent, but also for divalent ions such as calcium, magnesium, sulfate, nitrate, and phosphate [31]. In 1969, Evans and Accomazzo carried out two separate studies on the electrochemical demineralization process of water to remove ions by CDI. The results of this study showed that it is necessary for hydrogen adsorption and production of hydroxyl ions to a faraday reaction at the cathode. Also, the conditions created by the hydroxyl ion provide a suitable condition for the deionization of weak acid groups, and this leads to a demineralization reaction based on the ion exchange mechanism. During the reduction phase of the electrode, the applied voltage is reversed and this reduces the pH at the desired point, thus the ions removed in the deionization phase are separated from the electrode. Based on this mechanism, they believed that in order to achieve the demineralization cycle, the voltage must be reversed during the reduction phase, and they also believed that the salt removal efficiency depended on its surface concentration [32]. In 1969, Murphy conducted a study on the surface properties of carbonaceous materials before and after the reaction used to make the electrode. They investigated the effect of concentrated nitric acid and sulfuric acid on the process efficiency and reported that the response of the cationic electrode was similar to that of a selective ionic membrane. The cause was the formation of carboxyl groups by reaction with acid on its surface [33]. What has been discussed since the early 1960s about the candid properties of electrodes, with an emphasis on carbon-cathode electrodes, has become obsolete. It has been found that storage on electrodes absorbs ions into an area called the Electrical Double Layers and therefore, carbon-based electrodes can be used to make both the cathode and the anode, and it was also found that the Faraday reaction is not particularly important in the CDI process [34]. In 1970, Johnson et al. presented a major revolution in the science of capacitive deionization. They considered it unnecessary to assume a Faraday reaction that could occur in the space between a solid and a solute and cause the electrode to break down [35]. Also, according to operational efficiency, this reaction is not necessary when the current flux is in the main path of the capacitor [34,36]. Johnson et al. introduced the real concept of the electrochemical demineralization reaction, formerly known as the Potential-Modulated Ion-Sorption theory, with the Electrical Double Layers theory (EDL), which is an acceptable mechanism for ion removal using asymmetric conditions in the charge and discharge cycle. The use of these operating conditions is very important because this theory shows that by using unequal times in the charge and discharge cycle or optimizing the cell voltage during the charge phase, the system performance can be improved without changing the polarity. For the first time, an economic feasibility study was conducted in terms of cost and showed that a capacitive method is cost-effective if it is possible to build a stable electrode [35]. In 1971, Johnson and Newman made the ion absorption capacity of electrodes dependent on the electrical capacity of the double layers, the available contact surface of the electrodes, and the voltage applied to the cell, by making a cylindrical carbon electrode made of porous carbon and assuming that the electrical double layer capacity was constant [37]. In 1972, Soffer and Folman conducted an experiment to measure the effect of an electric double layer on the porosity of electrodes. Their results showed that the difference between the adsorption capacity of porous carbon and mercury was very small and concluded that even very small porous spaces at the nanoscale (0.5–3 nm) could be useful for ion adsorption [38]. In 1978, Oren and Suffer introduced the four-function electrochemical parametric pumping cycle theory, which will be explained in detail in the following sections. They used this method to obtain a correct separation of salt from a solution of water and salt based on an electrical double layer in porous carbon electrodes [39]. In 1983, Oren and Suffer investigated the amount of ion adsorption in the electrical double layer by constructing and introducing a closed-state system using the four-factor electrochemical parametric pumping cycle theory [40].

About 20 years after the advent of the capacitive deionization method, the major research conducted in object with the theoretical discussions of this method was not completed and until then, no materials could be found that use a good stability state to make the electrode. This led to a 25-year hiatus in the development of this method [7]. Acceptable CDI electrodes must have properties of high specific capacity, high conductivity, low resistance to ion transfer in the ion absorption phase, low cost of materials and fabrication, and chemical and electrochemical stability in thousands of charge and discharge cycles [22,41,42].

In the 1990s, Farmer et al. Introduced a new deionization system that used carbon aerogel as the electrode material, and this led to a dramatic breakthrough in capacitive desalination. The properties of carbon aerogel were very important due to its low density, high electrical conductivity, and porous holes at the nanoscale [43,44]. From 1990 to 2012, most research was conducted on capacitive deionization technology focusing on the construction of electrodes with structural characteristics of porosity space, which is a combination of interconnected networks of large porosities (mesopores and macropores) that are connected to the space of smaller porosities (micropores) [7,25]. Large porosities facilitate ion transport and small porosities increase the adsorption capacity.

Most CDI electrodes are made from a combination of carbon powder particles, a polymeric binder, and usually a conductive like black Carbon. These mixtures are spread on a current conductor (metal or graphite plates), which are also distributed mechanically (gluing and drying) to produce an electrode film of carbon particles [7]. Research has been conducted to use materials other than polymers as binders, including porous monolithic materials [45], carbon felts and fabrics [46,47], and Interwoven Array of Porous Fibers [48]. The results of this research showed that the electrode structures were more expensive than polymer binders.

The results of many research works led to the introduction of carbon as a base material for making electrodes in CDI applications and energy storage.

Development of new materials such as activated carbon [49,50,51], aerogel carbon [23,52,53], Ordered Mesoporous Activated Carbon [16,54], Carbon Nanotubes [55,56], Microporous Carbide-Derived Carbons [45,57], Modified Activated Carbon Cloth [17,58], Activated Carbon Nano-Fiber [59], Graphene-Based Material [60], rgo-sno composite electrode [61], carbon black [62] has been carried out to make electrodes for the past two decades.

Although new materials have been developed to introduce electrodes in the capacitive deionization process, one of the most common and suitable is activated carbon, which is also economical to manufacture [41,63,64].

A new type of desalination called Membrane Capacitive Deionization was developed and introduced in the last decade [65,66]. In this system, two ion exchange membranes are added to the capacitive cell, in which the anion exchange membrane is installed in front of the positive electrode and the cation exchange membrane is installed in front of the negative electrode. The purpose of this membrane is to change the direction of the electrodes during saturation to regenerate them, in which case the ions are removed from the electrodes. At this stage, the phenomenon of adsorption of ions with opposite charge occurs naturally, which causes a longer regeneration time, and to solve this problem, membranes are used on the electrodes to increase energy efficiency [67]. In 2013, Zhao et al., Conducted a study on membrane cells that showed that under constant current operating conditions, the output concentration was more uniform and more controllable than at constant voltage conditions. However, the problem with membrane cell design is that in addition to the extra cost of making and adding membranes, due to the deposition of these membranes, cell maintenance is difficult and their cost increases [27]. During the regeneration of the capacitive cell, a current is generated that can be stored and used in the next desalination cycle. AlKuran et al. [63] presented an auxiliary reduction circuit that transmits energy from a capacitor cell to a super capacitor by an indicator and showed that 50% of the energy required in the desalination step can be reduced by this circuit [68]. Landen et al. (2013) used this energy by directly connecting two electrodes at the same time, in which case only 25% of the energy is reduced [69].

## 3. Importance of the CDI Method

Based on human needs and technological advances, up to now, many methods for water desalination have been researched on and introduced. However, for many reasons, a small number of them have been able to have industrial and practical applications. The two most widely used desalination methods presently are Reverse Osmosis (RO) and Multi-Stage Flash Distillation (MSF) [70]. Now, a key question arises that given the availability of these methods, what is the reason and importance of using the capacitive method?

**Figure 2:**
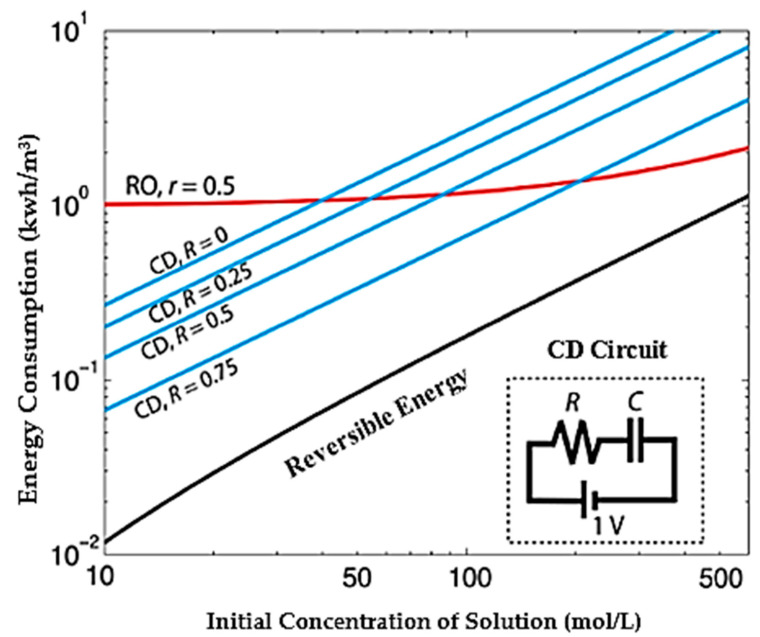
Estimation of energy consumption for reverse osmosis desalination and capacitive deionization method. Reprinted/adapted with permission from Ref. [25]. 2013, Suss, M.

The reverse osmosis method with the largest share of water desalination in the world uses mechanical energy and, by applying pressure to saline water and passing through a semi-permeable membrane, causes desalination of water which for each cubic meter of fresh water requires from 4.5 to 6 KW of energy [36,71,72]. In the thermal method, based on the principles of phase change and the use of thermal energy causing water vapor and then compressing it to produce desalinated water, the energy required in this method for each cubic meter of fresh water is between 5 to 10 kWh [73]. Figure 2 shows a comparison of the amount of energy consumed for the two methods of capacitive and reverse osmosis [25]. Assuming that the reverse osmosis method is capable of reducing 50% of water and by calculating the best working condition for the reverse osmosis method, the amount of energy required by this method is incomparable compared to the case where the CDI method is able to reduce 75% of water [74]. In addition to high energy consumption, factors such as chemical excretion, noise pollution, greenhouse gases, and constant concentration of water output are among the important and unacceptable disadvantages of reverse osmosis methods and thermal methods [4,12,13].

The method of capacitive deionization by producing no harmful chemicals, without any membrane and with the least energy required is the new generation of desalination plants [15,16]. “CDI method Unlike other methods that separate water from small salt particles, here the tiny solution (tiny salt ions) are separated from the abundant molecules of water, this causes less energy to be used to remove salt” [75]. The energy consumption required in this type of process is from 0.2 to 1 kWh to produce each cubic meter of desalinated water [76]. Another advantage of the CDI method is the adjustment of the salt output concentration to the desired level, which can be achieved by adjusting the applied voltage or current, while with other methods, the output concentration of the desalination system is constant [20]. Also, according to the principles of desalination, the CDI method can be used from very large industrial dimensions up to domestic applications [25]. “Also, since the CDI method does not require high pressure and temperature and is able to remove salt at a pressure lower than the osmosis pressure at an operating room temperature, it is possible to make plastic parts from it. This reduces the initial cost of construction and also prevents the formation of sediment in parts of the device, which are among the limitations of reverse osmosis methods and thermal methods. Therefore, this method is proposed as an attractive method to remove salt from water with high sediment. Conventional deionization methods suffer from the basic problem of high salinity residues in the deionization output and the production of secondary chemical waste, which is very promising to overcome this problem using the capacitive method, which is referred to as a green and economical method [77]. CDI method with no sound production can be a good option for desalination of water in homes and residential environments’’.

## 4. Geometric and Operational Methods for Testing and Evaluating a CDI Cell

### 4.1. System Structure

In addition to the many advances in theory and research to make the proper electrode over the past few decades, various methods have been developed for the construction of the CDI cell and the placement of the electrodes. Based on the method of operation, two methods were introduced by researchers, one of which is similar to the methods used in the testing of Supercapacitors which is based on laboratory electrochemical analyzes related to the Faraday reaction, three electrodes (working, counter, and reference electrode) are being used. In another method, which is performed for water desalination tests, two carbon electrodes are used to fabricate the cell.

### 4.2. Designs Used in the Capacitive Method Based on Cell Construction with Two Electrodes

**Figure 3:**
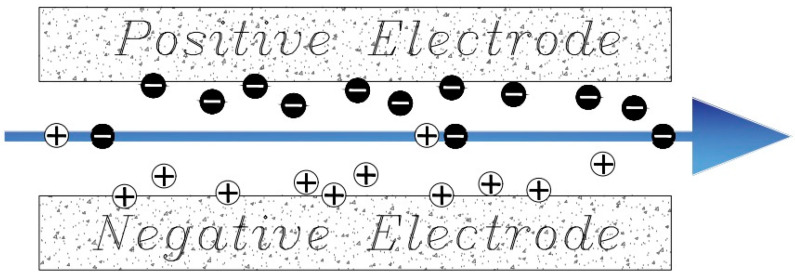
Flow-By method in water desalination by CDI method.

Similar to electrochemical capacitors or supercapacitors, a CDI unit consists of two electrodes. One of the most common arrangements of CDI cells is the placement of two carbon electrodes in parallel and at a very short distance from each other. The electrodes are made of the same material and have the same dimensions and size. Based on the direction of the water flow towards the electrodes, the geometry of this design is divided into two terms: Flow-By and Flow-Through [34,78]. The most widely used Flow-By (or Flow-Between) method (Figure 3) uses a 5 × 5 cm^2^ or 10 × 10 cm^2^ on a laboratory scale. The electrodes are placed either in a compressed film or as a coating on a conductive plate [79,80]. The flow of water in this type of electrode passes through a space of about 1 mm between the two electrodes and is perpendicular to the direction of the electric field [75]. This method is most used in evaluating salt adsorption behavior. To increase the volume of water desalination, several separate cells can be integrated together [81,82]. In this method, there are different paths for water to pass between the two electrodes. In another method, water enters through the hole in the middle of the cell and exits radially from all four sides of the cell. This radial method is very desirable in units where several cells are connected [82].

**Figure 4:**
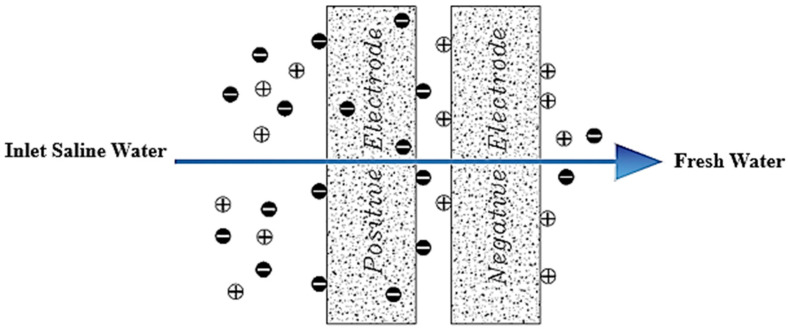
Flow-Through method in water desalination by CDI.

The famous geometric method developed by Newman and Johnson in 1970 for the purpose of water desalination is known as Flow-Through. In this method, the direction of water flow is perpendicular to the electrodes and parallel to the field direction (Figure 4). In this method, water is passed through the electrodes with pressure and due to the reduction of ion transfer time from saline solution to the electrodes, the rate of deionization is higher than the previous method, but the size of the pores and the effective surface of the electrodes must be increased. Also, due to the structure of this method, no channel is required for the passage of fluid and it reduces the ohmic resistance [78,83].

**Figure 5:**
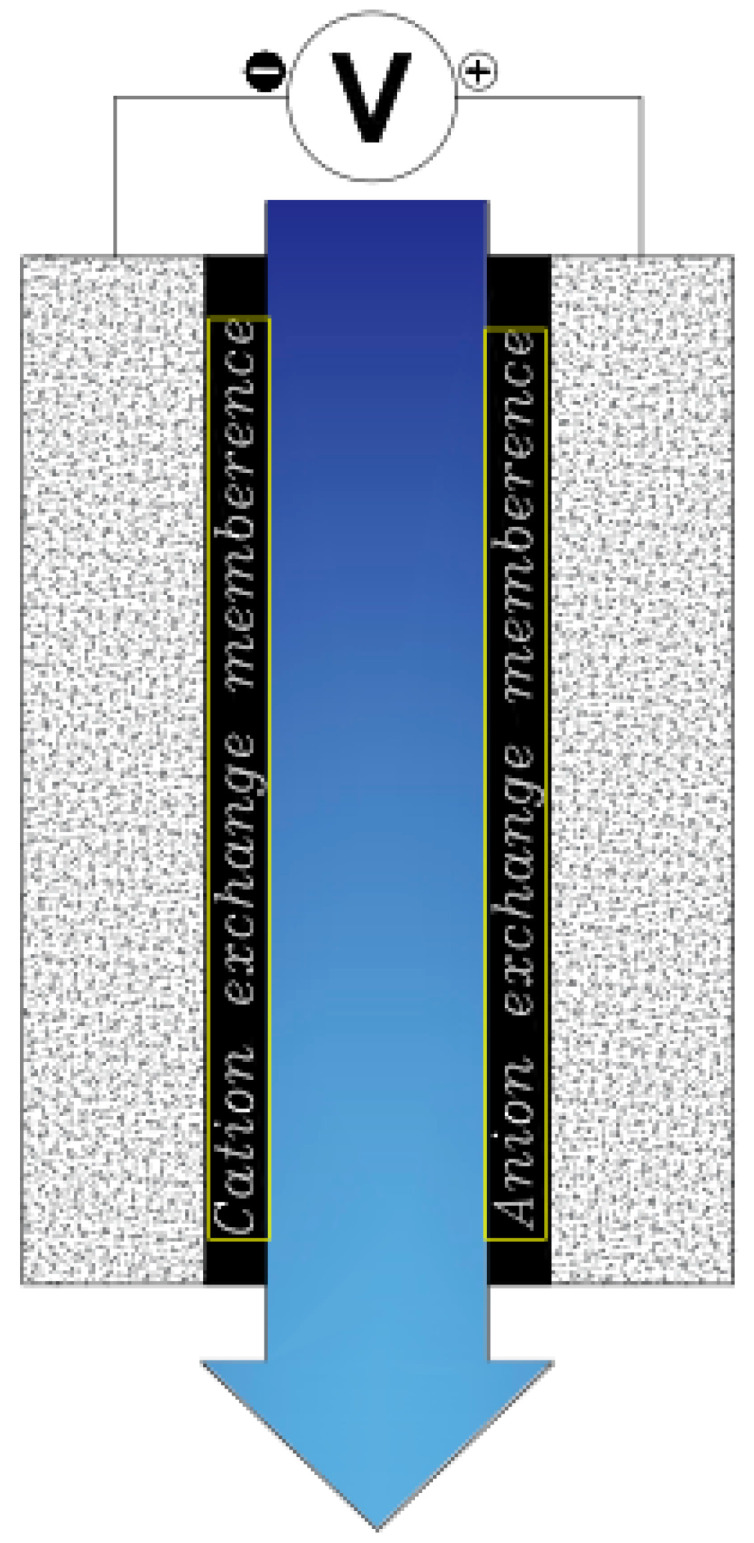
Ion exchange membrane in CDI cell.

Another change made by Lee et al. in 2006, to the structure of a capacitive cell was the addition of an ion-exchange membrane at the front of each electrode (Figure 5). This type of cell is known as MCDI [66]. Membranes can be added in two ways. In the first case, the membrane is installed completely separate from the electrode [34]. In the second case, the membrane is placed on the electrode as a coating [84]. Adding these membranes can selectively separate the desired ions from the solution [19]. Some research has reported an increase in charge efficiency due to the addition of these membranes [85,86].

**Figure 6:**
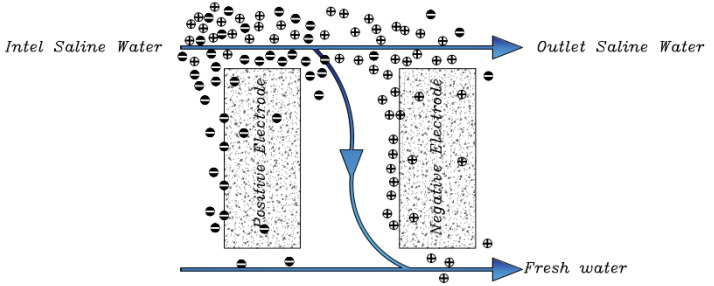
Installed electrostatic ion pumping in a CDI cell.

Another method known as electrostatic ion pumping has a completely different flow path for freshwater and condensed salt water [87]. The operation of this method is opened and closed by double alternating valves and controls the outlet flow [75]. The primary saline water is pumped from the top of the cell, and after passing through the electrodes, salt ions are absorbed and fresh water is generated at the bottom of the channel (Figure 6). After saturation, the electrodes are regenerated by a short circuit. During the regeneration phase of the electrodes, the water flow path can be reversed, which causes a saline water flow at the top of the cell, so the advantage of this method is that fresh water and saline water flow can exist simultaneously [36]. In other methods, currents are generated intermittently.

**Figure 7:**
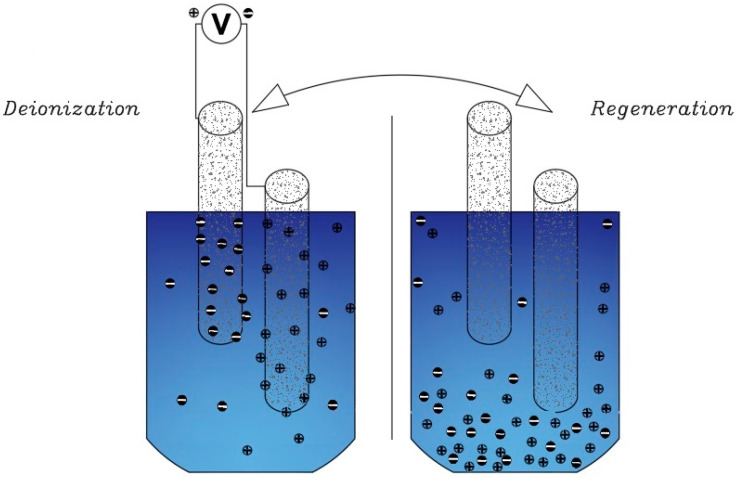
Wire electrode in a CDI cell.

A new method known as (Wire CDI) consists of anode and cathode interchangeable electrodes [81]. The main purpose of this project is to prevent the passage of fresh water and salt water through a single channel during the whole time of water desalination. The principle of operation of these electrodes is that the cathode and anodic electrodes are placed in a tank or path and the charging phase begins. This process continues until the electrodes are fully charged, and after saturating the electrodes, remove them from the tank and place them in a separate stream or tank for regeneration, and the discharged electrodes enter the tank from the previous step (Figure 7). Continuity of this cycle prevents the desalination process from being interrupted [75].

**Figure 8:**
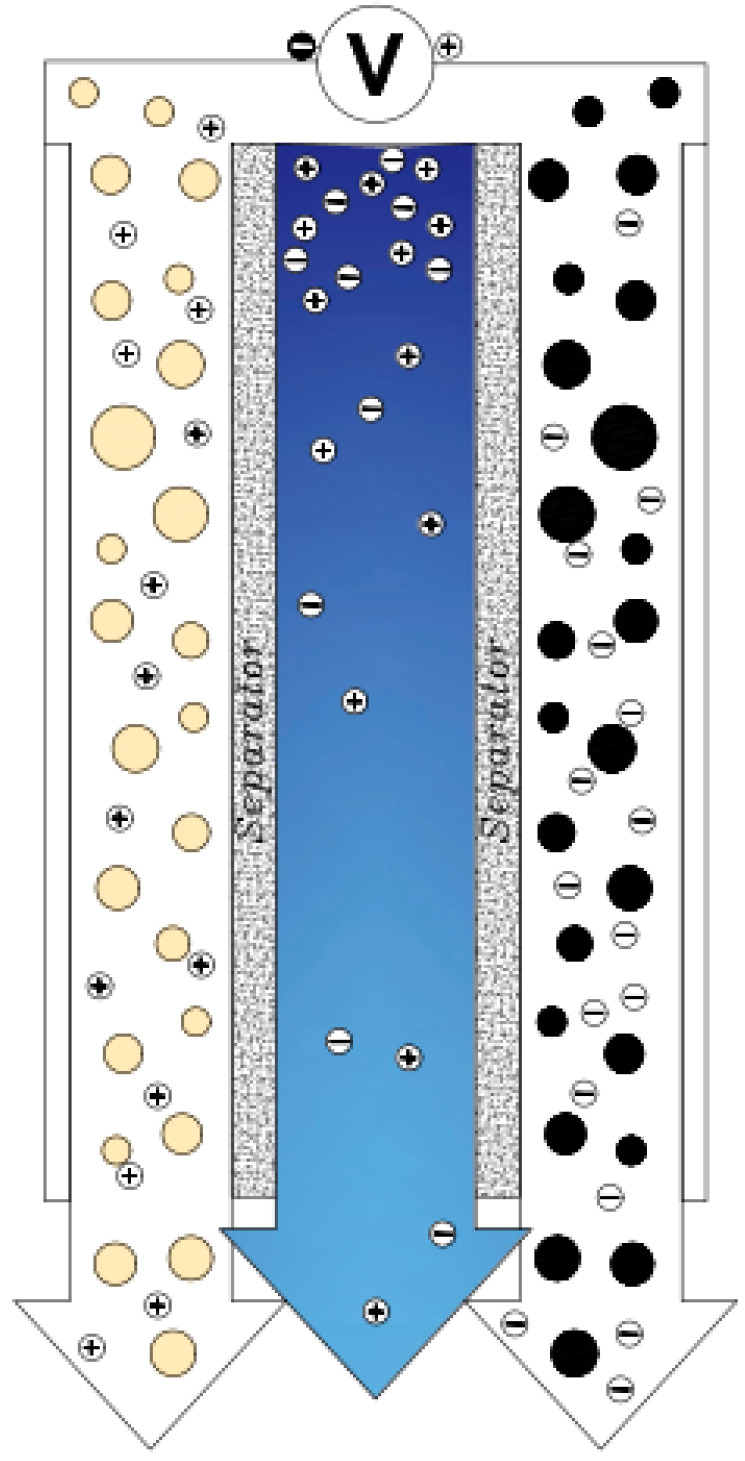
Flow Capacitive Deionization electrodes in CDI cell.

Another method that works like electrochemical current capacitors is known as Flow Capacitive Deionization (FCDI) [23]. In this method, instead of solid carbon, a type of slurry carbon is used in the role of an electrode and the flow of salt water and slurry carbon are in contact with the current (Figure 8). As the slurry is charged, the salt particles are absorbed into the Electrical Double Layers. Slurry carbon with ions is adsorbed by a filter (about 100 μm) separated from the saline stream to produce desalinated water [88]. The advantage of this type of electrode over the common type is that in this method, the reduction phase of the electrodes is eliminated, and this has led to continuous and uninterrupted desalination. Another advantage of this method is that water with high salinity can also be deionized [23].

## 5. Method of Performing CDI Tests

**Figure 9:**
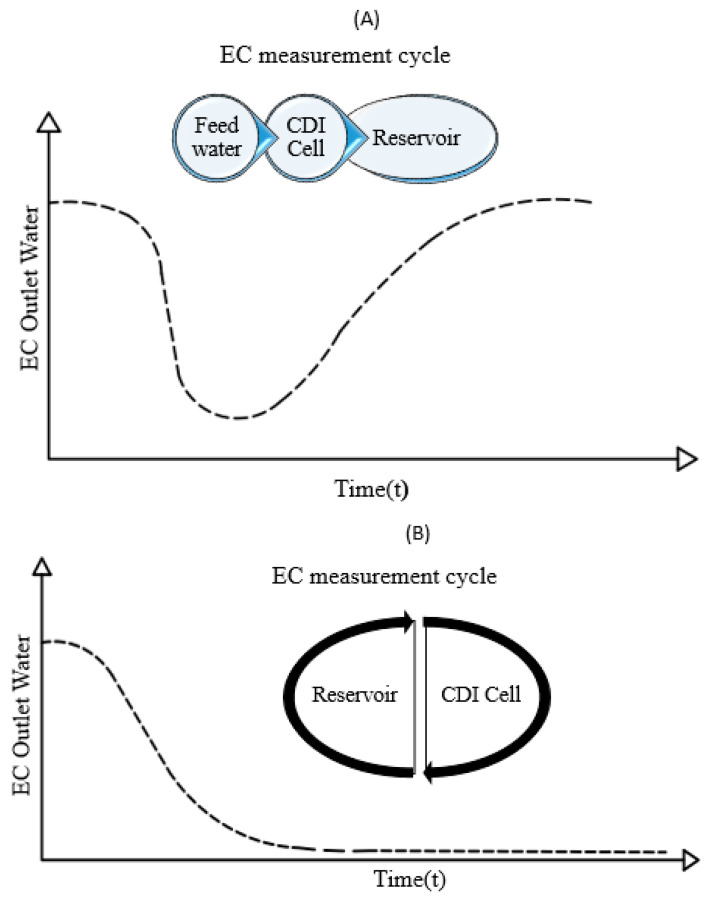
Schematic of two designs used in CDI experiments: (**A**) Single-Pass (SP) method, (**B**) Batch-Mode (BM) method.

In all arrangements of electrodes to make a CDI unit to evaluate the performance of the CDI method in the water desalination process, tests are performed to measure the adsorption capacity of the electrodes, which requires measuring ion concentration changes over time. Depending on the location of the electrical conductivity measuring probe, two methods, Batch Mode (BM) and Single Pass (SP) are introduced [89]. In the SP method, water flows from a reservoir into a CDI cell with a certain degree of salinity, and after passing through the cell, its salinity (Electrical Conductivity (EC)) is measured directly [57,90]. The installation site is the electrical conductivity meter after the desalination unit and before the outlet tank. In this method, the amount of salinity measured by the output current is initially reduced rapidly to the initial amount of salinity in the cell. In this method, the outlet water tank can either be completely separated, in which case the water is stored in another tank after leaving the cell or returned to the original tank, in which case the size of the tank is such that the salinity changes at the inlet and outlet are less than 1%. In this method, the amount of removed salt molecules is calculated by calculating the area below the output concentration versus the time diagram (Figure 9A). The SP method is very effective for places that use a large source of saline water such as the sea or wells [20,91].

Another method, known as BM, is to place an electrical conductivity measuring rod inside a water collection container [50,54,79]. In this method, the size of the water collection vessel should be small enough to measure the salinity with high accuracy. In this method, the amount of salinity decreases slowly and reaches a constant value after the electrode capacity is completed (Figure 9B). In this method, to calculate the number of ions removed, the difference between the initial and final salinity is measured and multiplied by the total volume of water in the system. This method is used in places where the amount of water in a tank is constant, such as well water [57,81].

A general conclusion comparing the two methods is that the BM method is simpler to analyze than the SP method. However, the weakness of the BM method is that at any given moment the balanced salt concentration is measured as the difference in concentration in the tank in each experiment, and this value is not comparable to any number. Therefore, in the BM method, it is not possible to compare the absorbed salt data in different concentrations and voltages, and the reason is that with increasing voltage, the intensity of salt absorption increases and theoretical and laboratory analysis of this method is impossible. This problem is not seen in the SP method because in this method all the characteristics (salt concentration, voltage, etc.) can be measured at any time. Another advantage of the SP method is that it is more practical in terms of development on an industrial scale [34].

## 6. Theory and Dominant Principles Capacitive Deionization Method

**Figure 10:**
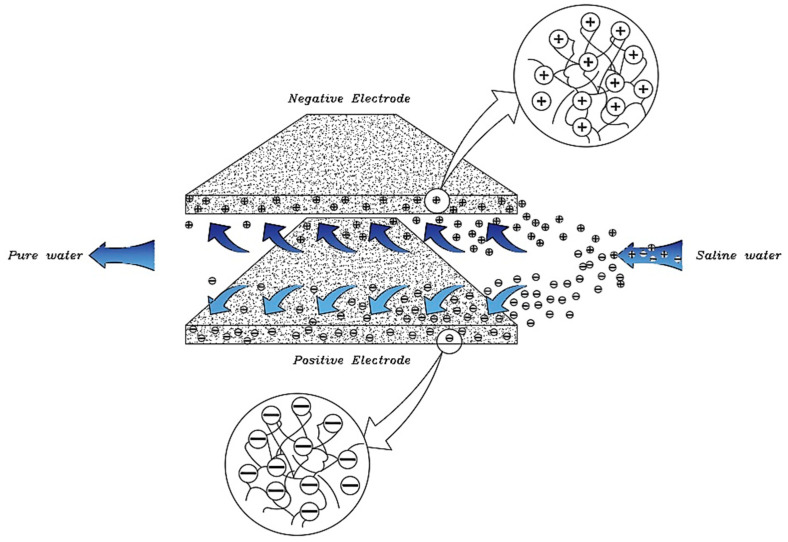
Sorption phenomenon in water desalination process by CDI method.

The CDI process of removing ions and charged molecules that are in the form of ions has taken the term deionization. According to the definition of a capacitor, which is a device consisting of one or more pairs of electrodes or a charged plate and potential is applied to them to create positive and negative poles, the salt removal method is called Capacitive Deionization. In principle, the mechanism of the CDI method is based on the principles of electrochemistry, and since the capacitor is an electrochemical cell, it facilitates the sorption phenomenon. The sorption phenomenon is based on the separation of particles of a liquid by the external or internal surface of solids and other liquids. The adsorption phenomenon includes two processes of adsorption (surface sorption) and absorption (internal sorption). Adsorption or surface sorption indicates the adhesion of ions, atoms, or molecules of a liquid or dissolved solids to the adsorbent surface (electrode), which creates a film of adsorbent on the adsorbent surface (Figure 10). However, in absorption or internal sorption, liquid or solid ion particles dissolved in the adsorbent penetrate and dissolve in it. Therefore, the positive feature of the CDI method compared to other methods that use the sorption process for deionization is that by using special electrodes (carbon) and with the help of simultaneous electric force, the adsorption and desorption process is used and has the ultimate benefit in increasing the ion adsorption capacity [92]. Table 1 lists the theoretical and conceptual developments of the CDI system and their progress reported in the literature.

To understand the behavior and function of the electrode in a CDI cell, it is important to understand the electrochemical knowledge of this phenomenon. For ions to be absorbed by the electrodes, the importance of the applied potential being close to the reference potential of the electrode is very significant. Choosing the proper potential for optimal ion adsorption and minimizing the Faraday reaction is important and fundamental, and if this potential is not selected correctly, the amount of ion adsorption will not be optimal [104,105]. Since all electrochemical processes are associated with the oxidation-reduction phenomenon, the occurrence of the oxidation-reduction reaction at the carbon electrodes of the CDI cell is not unexpected. Because the optimal potential of an electrode depends on the zero-charge potential (PZC) of the constituent material of the electrode, by modifying and performing oxidation-reduction reactions of carbon materials, the optimal value of the carbon electrode potential can be obtained, thus improving CDI cell performance. This is done by applying a decreasing half-reaction in the positive electrode and tending its PZC value to a negative value and also applying an oxidation half-reaction on the negative electrode, its PZC value tends to be positive [34]. Another way to optimize the potential of the electrodes is to use an additional electrode (reference electrode), which can be increased by comparing the potential of this electrode, charging efficiency, and absorption capacity of the electrodes [63].

### 6.1. Electrochemical Reactions and Processes in the Relationship between Carbon and Electrolyte

In addition to the extensive research that is being conducted on the ingredients for making electrodes, the discussion of theory and chemical events has also been widely considered to improve the performance of the CDI system. According to the mechanism of the capacitive method, by applying a voltage difference between two electrodes, ions absorbed and these particles are kept on the electrode by electrostatic force, and by removing this voltage difference, ions are separated and the electrodes are regenerated. This causes complex and varied reactions in the space between the electrodes. Some of these reactions play the main role in the CDI method, and some have effects that reduce or increase the performance of the CDI system. In general, all reactions considered in the CDI phenomenon are divided into two main groups: (1) Faraday processes and (2) non-Faraday processes [106]. The difference between the two methods is that in the first group, electrons participate in electrochemical reactions with carbon/electrolyte reactants and by-products, or in the electrolyte phase [34].

### 6.2. Faraday Reactions

**Figure 11:**
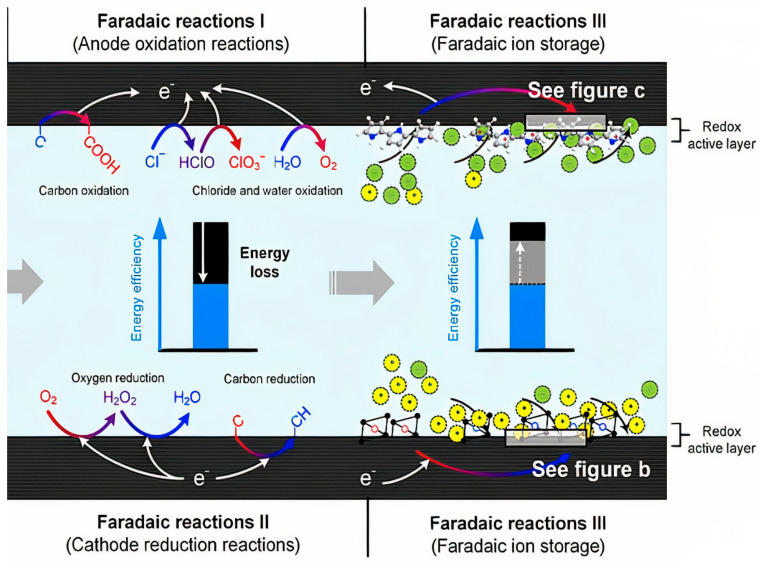
Schematic of three types of Faraday reactions in the capacitive deionization process. (Faradaic reaction I) Anode oxidation reaction, (Faradaic reaction II) Cathode reduction reaction, and (Faradaic reaction III) Faradaic ion storage. Reprinted/adapted with permission from Ref. [98]. 2018, Zhang, C.; He, D.; Ma, J.; Tang, W.; Waite, T.D.

Faraday reactions were first investigated as a phenomenon for the removal of ions from drinking water [35]. However, it is now clear that these reactions can have both advantageous and disadvantageous effects on CDI performance. Some of the Faraday reactions that occur in CDI can cause the negative performance of the electrode, energy efficiency, useful life of the electrode, chemical byproducts, or fluctuations in pH of the water, while it can also have positive effects such as increased false capacity or production in the time taken to absorb ions [107,108,109]. Faraday reactions are generally divided into three categories (Figure 11) [98].

The first category, known as anodic oxidation reactions (Faradic reaction I), causes oxidation at carbon electrodes, oxidation of chloride, oxidation of water, and other contaminants such as minerals and non-minerals. The most important thing that happens by oxidative reactions is its effect on the carbon electrode, which has destructive effects on the porosity structure as well as a decrease in the electrode mass, which reduces the life of the electrode and the poor performance of the CDI unit [18,110].

The second category is based on the grouping of the cathodic-reduction reaction (Faradic reaction II). Most of this reaction is related to the oxygen reduction reaction. This causes an asymmetric distribution of the anode and cathode potentials in the electrochemical reaction, which in turn intensifies the anodic oxidation reaction. The oxygen reduction reaction produces by-products such as hydrogen peroxide (H_2_O_2_), the beneficial effect of which is to disinfect water or reduce pollution caused by organic matter. In addition, the cathodic-reduction reaction can separate heavy metals from the water, causing the electrodes to precipitate [108].

The third category is Faraday ion storage (Faradic reaction III). In this process, the effective lateral capacity to store ions through reversible reduction-oxidation reactions replaces the electrostatic storage in the space between the electrode and the electrolyte in the Electrical Double Layers [107,111,112].

Due to the novelty of the ability to improve the performance of the CDI system and get the most out of this method, extensive research in all areas and factors affecting this method is still ongoing, given that the Faraday reaction initially was not considered a very effective factor in this method. Today, in order to increase the efficiency of the CDI cell, it has been studied in detail by researchers, and mentioning these activities in detail is beyond the scope of this text. For more information, refer to [113,114].

### 6.3. Non-Faraday Reactions

Removing impurities from liquids and obtaining a product with a final and acceptable purity has long been considered by various industries. Some industries seek to remove contaminants from production and factory waste, while others seek to purify the manufactured product. Therefore, various methods and theories have been proposed for this purpose. Much research has been conducted on the use of electrochemical capabilities to absorb ions and transfer them between an electrolyte medium and a charged surface, which led to the introduction of the Electrical Double Layers theory. This concept has been widely defined and used in electrochemical devices such as batteries, supercapacitors, electrical appliances, and deionization systems [115,116].

### 6.4. Electrical Double Layers Theory

**Figure 12:**
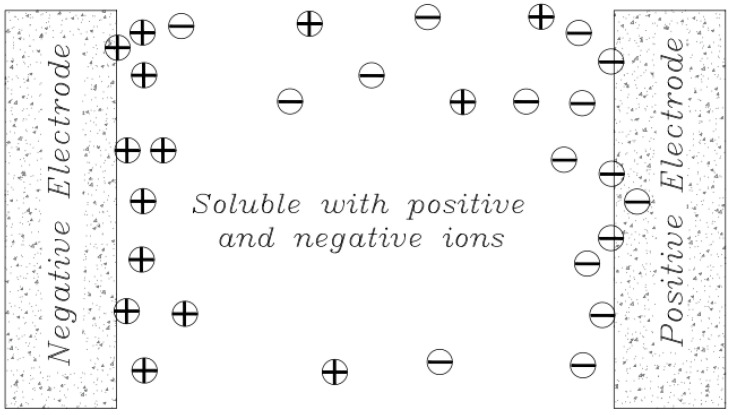
Schematic of ion transfer and principles of Electrical Double Layers formation and charge distribution in a system consisting of two electrodes.

The original definition of an Electrical Double Layer meant that for a common surface length between the electrolyte solution and the solid phase (carbon electrode) that could be charged separately, it charged as soon as an additional charge was applied in one phase (electric charge on the carbon electrode). The charge generated by this charge is neutralized in the other phase (electrolyte), where the total amount of electron charge at this level (electrode-electrolyte contact plate) will be zero, and this will neutralize the Electrical Double Layers. The concept was first introduced by the German physicist Herman von Helmholtz in 1853 and was later modeled in 1913 by Louis Georges Gouy and David Chapman. They hypothesized that the surface charges are induced by an external electric field to the body, and when the conductor is placed in an electric solution, an electric current (I) is created by applying an external electric field (E) that follows the equation I = σE. In this equation, σ represents the conductive electrical conductor [49]. By applying this field and creating a current, the electrolyte-soluble ions reach the surface of the body, and therefore in a thin layer of the surface around the body, positive charges are placed on one side and negative charges on the other. In fact, an equal charge but with different signals is induced on the surface of the body, which absorbs oppositely charged ions in the electrolyte solution. As a result, a bipolar layer of ions is formed near the solid-liquid surface (Figure 12).

**Figure 13:**
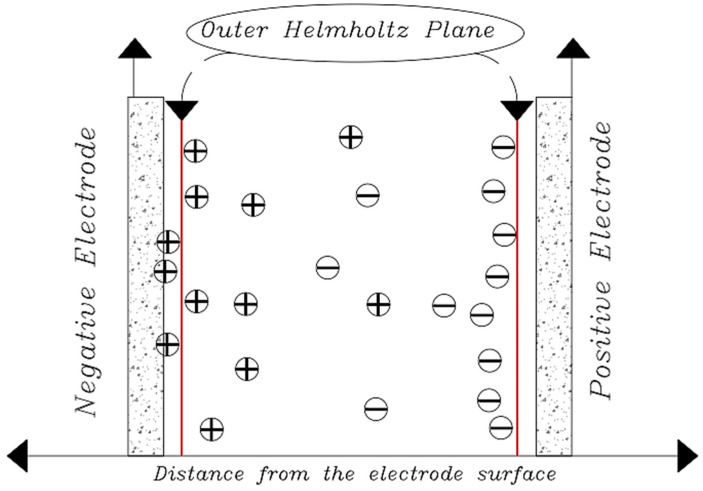
Load distribution in a system with charged electrodes.

If the principles of the Helmholtz model are to be followed, for each electron moving from a charged electrode to the opposite electrode, one cation must move to the cathode (assuming the ions are monovalent) to neutralize the negative charge and one anion to the anode to neutralize it. The electric charge will move positively, resulting in an ideal state for the removal of salt ions between the two electrodes in the channel, which is the main purpose of the CDI method, in which case the charge efficiency is close to 1 [89]. In a CDI cell composed of a porous electrode structure where the ions are not concentrated exactly on the plate after the electrode surface, a unit efficiency cannot be achieved and the theory of the electrical double layer cannot be accurately explained by the Helmholtz model in a capacitive cell [7,117]. To reveal the ion distribution very accurately, Gouy–Chapman introduced the concept of Electrical Double Layers theory, developed the diffusion layer model, and modeled it theoretically based on mechanical and mathematical principles. According to their theory, the diffusion layer, or the Gouy–Chapman (Debye) layer, is separated from the electrode surface by a compact layer, also known as the inner layer or the Helmholtz layer [118]. Their theory states that the thickness of the diffusion layer depends on the electrolyte concentration and the amount of applied potential. Applying more potential causes a higher charge density on the metal surface, which creates a stronger electrostatic force to absorb the charged ions, which causes the diffuser layer to contract, which in turn increases the electrode capacity [119,120]. Their theory showed that by moving away from the electrode surface and moving towards the electrolyte, the potential decreases exponentially (Figure 13). There is no exact information to show the thickness of the diffusion layer, but it can be estimated by a longitudinal characteristic in which the voltage and concentration of opposing or non-identical ions decrease by a factor (e ~ 2.7) known as the Debye length:

(1) $$\lambda_{d}=\sqrt{\frac{\varepsilon_{0}\varepsilon_{D}\cdot \mathrm{RT}}{F^{2}\cdot \sum^{\;}Z_{j}^{2}C_{j}}}=\sqrt{\frac{\varepsilon_{0}\varepsilon_{D}\cdot \mathrm{RT}}{2F^{2}\cdot J}}$$

$\lambda_{d}$ is the length of the Debye,$\;\varepsilon_{0}$ is electric constant, (8.85 × 10 ^−12^ F/m), ${\;\varepsilon }_{D}$ water dielectric constant (78.5 at a temperature of 25 °C) and J is the ionic capacity of the electrolyte. For a sodium chloride salt solution with a concentration of 10 mmol ionic strength, the Debye length is approximately 3.1 nm [34]. With a high accuracy, it can be assumed that the thickness of the diffusion layer is 2 to 3 times the length of the Debye [121].

A simple analysis for the distribution of salt ions in the Electrical Double Layers can be shown by the Nernst equation and the Boltzmann distribution:

(2) $$\ln\left[\frac{C\left(x_{j}\right)}{C\left(x_{i}\right)}\right]=\left\{\frac{-\mathrm{ze}\left[\left(\emptyset \left(x_{j}\right)-\emptyset \left(x_{i}\right)\right)\right]}{\mathrm{kT}}\right\}$$

Here C represents the ionic concentration of the particles in different states, ∅ applied voltage, Z charge or ion capacity, and KT/e is known as the thermal voltage at which k is the Boltzmann constant, e is the particle energy at the moment, and T is the thermodynamic temperature.

(3) $$c\left(x_{j}\right)=c\left(x_{i}\right)\exp\left\{\frac{-\mathrm{ze}\left[\emptyset \left(x_{j}\right)-\emptyset \left(x_{i}\right)\right]}{\mathrm{kT}}\right\}$$

Considering the application of the Poisson equation in electrostatic problems and derivation of the Gauss equation:

(4) $$\frac{d^{2}\emptyset }{{\mathrm{dx}}^{2}}=\frac{\rho_{f}}{\varepsilon }$$

In this equation, $\rho_{f=\sum^{\;}c_{i}z_{i}F}$ equals the charge density and ε is the de electric constant.

The boundary conditions in this system will be as follows:

$$\emptyset \left(x\to \infty \right)=0\;\emptyset \left(x\to d_{s}\right)=\emptyset_{0}-\Delta \emptyset_{s}$$

where d_s_ are equal to the thickness of the stern layer and ∅_s_ is equal to the voltage drop.

By moving from the electrode surface to the middle of the solution and combining the two Equations (3) and (4), we reach the famous Poisson–Boltzmann equation. For an ionic solution with a chemical capacity of 1:1 such as sodium-chloride salt, the Poisson–Boltzmann equation for the distribution of salt ions in solution would be as follows:

(5) $$\frac{d^{2}\emptyset }{{\mathrm{dx}}^{2}}=\frac{{\mathrm{Fc}}_{\infty }z}{\varepsilon }\sinh\left(\frac{\mathrm{ze}\emptyset }{\mathrm{kT}}\right)$$

By integrating from Equation (5), the charge density of the electric double plate is calculated as follows:

(6) $$q=\int_{0}^{\infty }\rho_{f}\mathrm{dx}=-\;\varepsilon \int_{0}^{\infty }\frac{d^{2}\emptyset }{{\mathrm{dx}}^{2}}\mathrm{dx}$$

According to the stated theory, as well as the results of another research [40,122,123,124], the number of ions adsorbed in the Electrical Double Layers depends on the amount of surface charge, the electrical potential applied to the electrode, and the conductivity of the solution.

**Figure 14:**
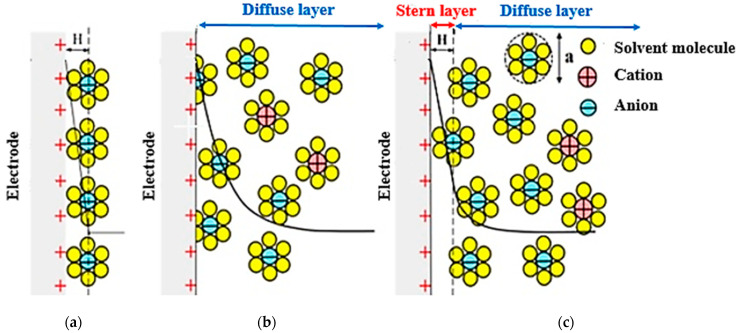
Schematic diagram of how to create Electrical Double Layers. (**a**) Helmholtz model, (**b**) Gouy–Chapman model, and (**c**) the Gouy–Chapman-Stern model. Reprinted/adapted with permission from Ref [62]. 2018, Baroud, T.N.

In 1924, Otto Stern combined the Helmholtz model with the Gouy–Chapman model, stating that some ions, as Helmholtz stated, adhere to the electrode and form a compact layer known as the Helmholtz or Stern layer, some of which, according to the Gouy–Chapman theory is placed as a diffused layer (Figure 14).

The thickness of the stern layer is similar to the hydrate radius of the ions adsorbed on the electrode and is usually between 0.3 and 10 nm [125,126]. 

In the Electrical Double Layers theory, the amount of potential in the stern layer is considered equal to the zeta potential, which is one of the most important electrical properties of the surface. The amount of zeta potential is determined by the surface chemistry of the particles as well as the pH of the solution [127]. Zeta potential is an effective factor in the process of electrical absorption. This potential is referred to as a plate potential in which the velocity of the solution is zero relative to the velocity of the particles (shear plate) The zeta potential is directly related to the Electrical Double Layers. As the zeta potential decreases, the thickness of the EDL decreases, and under these conditions, the absorption capacity decreases. There is a very clear and proven relationship between zeta potential and rheology [128]. Many materials exhibit varying degrees of zeta potential when exposed to liquids or water. The value of the zeta potential can also be considered as an approximation of the electrode surface potential.

Regarding the pH value, since it is a matter of desalination of brackish water, if the pH is in the acidic range, it has the greatest effect on desalination, because with increasing pH, the amount of zeta potential decreases, and consequently less salt is absorbed.

**Figure 15:**
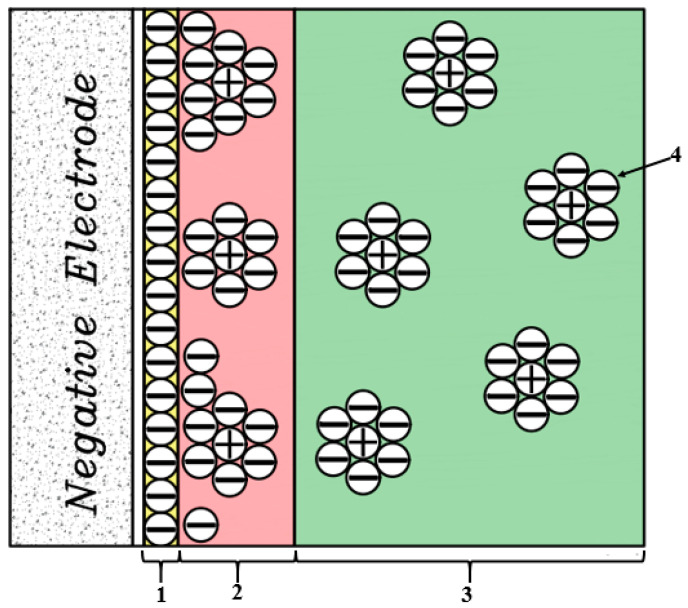
Stern extended model (Grahame model), 1: Inner Helmholtz layer, 2: Outer Helmholtz layers, 3: Diffusion layers, 4: Coated solvent ions.

In 1947, Grahame modified Stern’s model into two regions. He suggested introducing some ions closer to the electrode surface as the outer surface of Helmholtz and a layer of ions adsorbed on the electrode surface as the inner surface of the Helmholtz and described the electrical capacitance values of compact and scattered surfaces as two series capacitors (Figure 15).

The total capacitance of the Electrical Double Layers (C_dl_) can be calculated as the capacitance of two capacitors connected in series, one of which is equivalent to a stern (C_st_) or compressed layer and the other is equivalent to a Gouy–Chapman layer or (Cd) diffuser according to the following equation [119].

(7) $$\frac{1}{C_{\mathrm{dl}}}=\frac{1}{C_{\mathrm{st}}}+\frac{1}{C_{d}}$$

To estimate the stern layer capacity as a constant or as a function of the electrode voltage value, various models have been proposed by researchers, one of which is the following equation, which is expressed as a constant numerical stern layer capacity [115,129].

(8) $$C_{\mathrm{st}}=\frac{\varepsilon }{4{\pi d}_{s}}$$

In this equation, d_s_ is the thickness of the stern layer and ε is equal to the Dielectric constant.

The capacity of the diffusion layer, which depends on the amount of charge density, in the previous section was calculated based on the Gouy–Chapman-Stern theory, but the amount of this capacity can also be calculated according to the following equation for a monovalent electrolyte (such as NaCl):

(9) $$C_{d}=\frac{\varepsilon }{\lambda_{d}}\cdot \cosh\left(\frac{z\cdot \Delta \phi_{d}}{2}\right)$$

In this equation, Δϕ_d_ is equal to the potential difference across the diffusion layer, and λ_d_ is the value of the Debye length obtained from Equation (6).

Due to the structure of the consumable material, which should have a high porosity level, many models have been reported to describe the structure of the EDL both at the electrode surface and in the porosity space of the charged electrodes. Due to the porosity of the electrode material and the structure of the EDL, two models are proposed in most articles.

The first model is for materials whose porosity is much larger than the length of the die, which uses the Gouy–Chapman-Stern (GCS) theory. The second model is for materials with porosity less than the die length, which is based on the Modified Donnan theory.

### 6.5. Gouy–Chapman–Stern Theory

**Figure 16:**
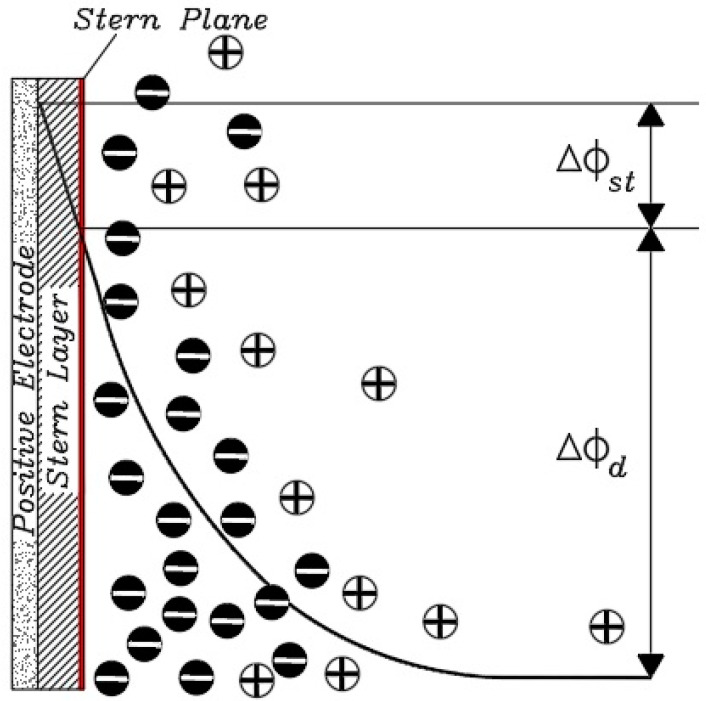
The potential difference in the Gouy-Chapman-Stern model in a non-overlapping porous space for the EDL.

For porosity greater than the length of the Debye, it can be assumed that the EDL in this space will not overlap, and therefore, the Gouy–Chapman model can be used for this type of porosity (Figure 16).

An important parameter that can be approximated by the Gouy–Chapman model and is one of the important characteristics in the CDI process to evaluate the performance of a capacitive system is known as charge efficiency (Λ), which is the ratio of salt removal versus the amount of charge applied to an electrode pair. When voltage is applied to a CDI cell, two phenomena can occur in the space between the two electrodes (in the diffusion region). One of these reactions consists of ions with the opposite sign to the charged electrode, which move towards the electrode and adhere to it. The second reaction also takes place with ions of the same name and all signs with the electrode, which move away from the vicinity of the electrode. Such neutralization of charge is not useful in the deionization process because for each electron that is exchanged between the two electrodes, only a part of it can be absorbed into the electrode plates and thus the amount of charge efficiency is reduced [130,131].

As previously described, by integrating the Boltzmann equation of distribution, the charge density in the diffusion layer can be calculated based on the potential difference in the diffusion layer (Δϕ_d_) [132].

(10) $$q=4\lambda_{d}C_{\mathrm{bulk}}\cdot \sin h\left(\frac{\Delta \phi_{d}}{2}\right)$$

In this equation, $C_{\mathrm{bulk}}$ is equal to solution concentration.

In 2010, Zhao et al. used the following equation to calculate the excess concentration of ions stored in the diffusion layer (density of salt adsorbed on the plate):

(11) $$\omega =8\lambda_{d}C_{\mathrm{bulk}}\cdot \sinh^{2}\left(\frac{\Delta \phi_{d}}{4}\right)$$

The following equation can be used to calculate the amount of charge efficiency in a capacitive system:

(12) $$\Lambda =\frac{\Gamma_{\mathrm{salt}}}{\Sigma }$$

In this equation Γ_salt_ is equal to the amount of salt absorbed in the electrodes of the capacitive cell as soon as voltage is applied to the system, also Σ is equal to the total charge transferred to the surface of the electrodes. 

The values of Γ_salt_ and Σ can be calculated both experimentally and theoretically. To obtain these values theoretically from Equations (11) and (12), we can use the assumption that the size of the two electrodes is the same and the potential value of the diffusion layer on both sides is the same:

(13) $$\Gamma_{\mathrm{salt}}=\omega \cdot a\;\mathrm{and}\;\Sigma =q\cdot a$$

In these equations, the value of a is equal to the specific surface area of the electrode in terms of square meters per gram of its constituent material.

Combining Equations (10), (12) and (13) leads to the introduction of a new form of charge efficiency equation in a capacitive system:

(14) $$\Lambda =\tanh\left(\frac{\Delta \phi_{d}}{4}\right)$$

Based on Equation (14) and the definition of the Gouy–Chapman theory, it can be concluded that the charge efficiency depends only on the potential difference in the diffusion layer (Δϕ_d_). To complete the Gouy–Chapman–Stern theory calculations, the amount of voltage applied to the cell can be attributed to the potential difference between the diffusion and stern layers. Assuming the symmetry of the two cell electrodes, the following equation can be used for this purpose [132].

(15) Vcell(2·VT)=|Δϕd+Δϕst|

In this regard, V_cell_ is the voltage applied to the cell, V_T_ is the thermal voltage = RT/F (at room temperature it is approximately equal to 25.7 mV), and Δϕ_st_ also shows the potential difference between the stern layers, which is directly related to the charge density. According to Gauss’s law, it is equal to:

(16) $$\sigma \cdot F=C_{\mathrm{st}}\cdot \Delta \phi_{\mathrm{st}}{\cdot V}_{T}$$

The stated calculations are valid and important for the conditions where the EDL is not overlapped and also the two electrodes are in a symmetrical state. That the Gouy–Chapman–Stern model calculates the salt adsorption capacity in the highest possible case but compared to other models, expresses the importance of the effect of surface area on the amount of salt adsorption.

### 6.6. Modified Donnan Model for Porous Spaces with Overlap in the EDL

In the previous section, the Performance of the Gouy-Chapman-Stern theory was discussed when the EDL does not overlap and the size of the porosities is greater than the length of the Debye.

Given the structure of the carbon materials used to make CDI cell electrodes, it is not acceptable to use this theory to express the complete behavior of a CDI system. The use of the Gouy–Chapman–Stern theory is not acceptable if the size of the pores and ion absorption pores is less than the length of Debye and there is also overlap in the EDL [130]. To solve this problem and study the behavior of a system that has an EDL overlap and the average pore size and porosity in its particles is less than the length of Debye and in the range of 1–2 nm, researchers suggest using the Modified Donnan theory [34]. The Modified Donnan model can explain the amount of salt absorption and energy storage in micro-porous fields (less than 2 nm) [20,91,133]. In this range, Donnan’s theory assumes that the electrolyte inside the carbon particles and the overlapping space of the double layer has a constant electric potential without change at different points.

Donnan’s modified model assumes that a non-electrostatic chemical adsorption energy causes ions to be deposited into carbon particles during transfer from the electrolyte, a phenomenon that can occur even when there is no electric charge [134]. Donnan’s modified model for the concentration of ion particles in the micro-porous space inside carbon particles can be shown by the following equation:

(17) $$C_{J.\mathrm{mi}}=C_{\mathrm{bulk}}\cdot \exp\left(-Z_{j}\cdot \left(\Delta \emptyset_{\mathrm{st}}-\Delta \emptyset_{b}\right)+\mu_{\mathrm{att}}\right)$$

**Figure 17:**
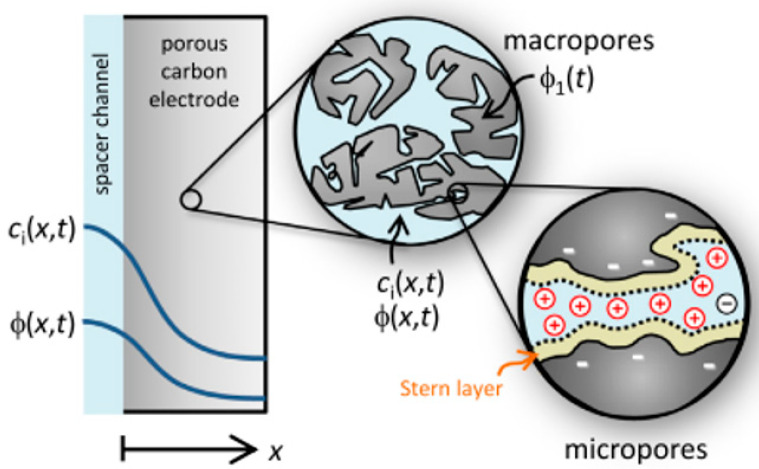
Donnan theory for the porous space of macro and microparticles with dual electrical layer overlap. Reprinted/adapted with permission from Ref. [34]. 2013, Porada, S.; Zhao, R.; van der Wal, A.; Presser, V. and Biesheuvel, P.M.

In this equation, similar to the Gouy–Chapman theory, it is assumed that a monovalent and symmetric salt (NaCl) is used. In this regard, $C_{J.\mathrm{mi}}$ indicates the concentration of ions in the porous space of the micro, $C_{\mathrm{bulk}}$, indicates the concentration of ions in solution, which is equal to the concentration of ions in the macropore (porosity greater than 2 nm), $Z_{j}$ is equal to the ionic capacity (equal to +1 for cations and −1 for anions), and $\Delta \emptyset_{b}$ is equal to the difference in solution potential, which is usually zero. Therefore, the potential difference in the stern layer ($\Delta \emptyset_{\mathrm{st}}$) is equal to the difference of the electron static potential of Donnan ($\Delta \emptyset_{D}$) between micro and macroparticles, or in other words, between the outside and inside of carbon particles (Figure 17). By summarizing Equation (17), the new equation can be rewritten as follows:

(18) $$C_{J.\mathrm{mi}}=C_{\mathrm{bulk}}\cdot \exp\left(-Z_{j}\cdot \Delta \emptyset_{D}+\mu_{\mathrm{att}}\right)$$

The equation obtained for Donnan’s theory is very similar to Equation (2), except that the equation uses the additional term μ_att_, which represents the non-electrostatic gravitational energy. Also, in this equation, instead of the local potential difference in Equation (2), Donnan potential difference is used. Note that in Equation (2), the calculation of the ion concentration at a distance (x) from the electrode surface is used and the integral equation is taken to obtain the charge density (Equations (10) and (11)). However, Equation (18) describes the total volume of the porosity space and does not need to be integrated, so the density of all the ions in the porosity space is equal to:

(19) $$C_{\mathrm{ions}.\mathrm{mi}}=C_{\mathrm{cation}.\mathrm{mi}}+C_{\mathrm{anion}.\mathrm{mi}}=2\cdot C_{\mathrm{bulk}}\cdot \exp\left(\mu_{\mathrm{att}}\right)\cdot \cos h\left(\Delta \emptyset_{D}\right)$$

Volumetric ion charge density in microporosity space (q_mi_) in mol/m^2^ can be obtained based on Equation (18):

(20) $$q_{\mathrm{mi}}=C_{\mathrm{cation}.\mathrm{mi}}-C_{\mathrm{anion}.\mathrm{mi}}=-2\cdot C_{\mathrm{bulk}}\cdot \exp\left(\mu_{\mathrm{att}}\right)\cdot \sin h\left(\Delta \emptyset_{D}\right)$$

The amount of ion charge density can be obtained based on the potential difference of the stern layer similar to that mentioned in Equation (16):

(21) $$q_{\mathrm{mi}}\cdot F=\Delta \phi_{\mathrm{st}}\cdot C_{\mathrm{st}\cdot \mathrm{vol}}\cdot V_{t}$$

In this equation, $C_{\mathrm{st}.\mathrm{vol}}$ is equal to the volume of the stern layer in volume (f/m^3^).

In the previous part of the discussion of the Gouy–Chapman–Stern theory, Equation (14) were used to obtain the amount of charge efficiency.

However, in Donnan’s modified model for SP systems where the salt concentration at the inlet and outlet is equal at one point in the Deionization cycle, the charge efficiency in the EDL according to Equations (19) and (20) is as follows [135].

(22) $$\Lambda =\frac{C_{\mathrm{ions}.\mathrm{mi}}-C_{\mathrm{ions}.\mathrm{mi}}^{0}}{q_{\mathrm{mi}}}=\tanh\frac{\Delta \phi_{d}}{2}$$

The number zero, in the numerator of the fraction of the above equation, indicates the amount of salt absorption when the applied voltage to the cell is zero.

According to Donnan’s modified theory for a CDI cell with two symmetrical electrodes, ($\Sigma_{F}$) is equilibrium charge and the amount of salt absorption (relative to the amount of salt obtained under zero voltage conditions) in terms of total mass for two electrodes in a CDI cell based on $q_{\mathrm{mi}}$ and $C_{\mathrm{ions}.\mathrm{mi}}$ are calculated according to the following equations [20,57,67,81]:

(23) $$\Gamma_{\mathrm{salt}}=\frac{1}{2}\cdot \frac{p_{\mathrm{mi}}}{\rho_{e}}\cdot \left(C_{\mathrm{ions}.\mathrm{mi}}-C_{\mathrm{ions}.\mathrm{mi}}^{0}\right)$$

(24) $$\Sigma_{F}=-\frac{1}{2}\cdot \;F\cdot \frac{p_{\mathrm{mi}}}{\rho_{e}}\cdot q_{\mathrm{mi}}$$

In these equations, $\rho_{e}$ is equal to the density of the electrode (mass per unit volume of the electrode), $p_{\mathrm{mi}}$ is the volume of the porosity space relative to the total volume of the electrode. In these equations, because the ionic concentrations are equal to the total cations and anions, the expression 1/2 is used to denote the total molecules of salt in the adsorption of a particular type of salt. Also, in these equations, the expression $\frac{p_{\mathrm{mi}}}{\rho_{e}}$ is equal to the volume of the porosity space in terms of grams of the weight of carbon powder made for the electrodes, which is mainly measured by the absorption of nitrogen gas. Similar to Equation (12) in the discussion of the Gouy–Chapman–Stern theory, the charge efficiency in the Donnan model is obtained from the ratio $\Gamma_{\mathrm{salt}}$ and $\Sigma_{F}$. A noteworthy point from Equations (22)–(24) is that assuming the capacitance of the CDI cell, the structure of the EDL at the cathode and anode is equal, with the only difference being the positive and negative signals. Also, the non-electrostatic attraction energy (μ_att_) according to this theory is equal for the anode and cathode. Finally, similar to what we observed in the discussion of the Gouy–Chapman–Stern model, the relationship between the applied voltage to the cell ($V_{\mathrm{cell}}$) and the potential difference in the Donnan layer ($\Delta \emptyset_{D}$) and stern ($\Delta \emptyset_{\mathrm{st}}$) will be as follows:

(25) Vcell(2·VT)=|Δ∅D+Δϕst|

## 7. Standardization of Parameters and Criteria for Measuring the Performance of a Capacitive Deionization System

To streamline reports and accelerate the progress of a system, it is necessary to standardize the information and results of activities related to that system. The field of capacitive deionization has been studied and tested by researchers in the last two decades with significant progress and the need to standardize will be the key to the progress of the industry’s leading activities. The definition of performance criteria became very important after the advent of different terms and the ability to properly compare the performance data of a CDI system. With the introduction of capacitive deionization, most of the reports by researchers were in the form of a decrease in salt concentration after applying a constant voltage to a transient fluid through the cell, which is known as a common indicator in desalination technology. However, using this parameter alone cannot indicate the good performance of the system or electrode. Many variables can have a direct or indirect effect on system performance and reduced deionization. Therefore, recognizing and unifying these variables and parameters will help researchers in developing and sharing information.

### 7.1. Salt Absorption Capacity

In 1972, Soffer and Folman introduced the term Salt Adsorption Capacity (SAC) as the first functional parameter in the deionization process. Salt adsorption capacity was used as the most important parameter to evaluate salt removal in CDI experiments [110,136,137,138,139,140]. This unit usually evaluates a period of deionization (the beginning of one cycle to the next), but it can also be used to compare the behavior of the cell to a half cycle (adsorption or desorption). To present the SAC results, it is not possible to note at the beginning of the electrode construction and the initial work cycles that the electrode is not yet fully saturated, and the results of this parameter are acceptable when the value is fixed from one cycle to another. The SAC value is reported as the mass of salt absorbed by weight (mg/g) or volume (mg/ml) relative to the weight or volume of the electrode material [78]. In research work, SAC is mostly used by weight and the weight intended for electrodes is in dry conditions, which includes all materials used to fabricate electrodes such as porous material powder (usually activated carbon), adhesives, porous materials, and other additives for Improving the quality of the electrode [141,142]. As described in the previous sections, the solution to be deionized passes through the cell in two ways, the SAC calculation for each of which is different. In the SP method, where the amount of deionization is measured at the cell output, the SAC value is calculated as follows [143].

(26) $${\mathrm{SAC}}_{\mathrm{sp}}=\frac{\mathrm{The}\;\mathrm{amount}\;\mathrm{of}\;\mathrm{salt}\;\mathrm{removed}\left(\mathrm{mg}\right)}{\mathrm{Electrode}\;\mathrm{mass}\left(g\right)}=\frac{\dot{Q}\cdot \int^{\;}\left[C_{i}-C_{o}\left(t\right)\right]\mathrm{dt}}{m_{\mathrm{elc}}}$$

In this equation, $m_{\mathrm{elc}}$ represents the total mass of the electrode, $\dot{Q}$ the rate of water supply into the cell, $C_{o}$ and $C_{i}$ the initial concentration and the output concentration of the cell. Therefore, according to this equation, the amount of absorbed salt is obtained by integrating the time of change of salt concentration at the input and each period of the deionization cycle by multiplying the amount of feeding intensity.

In the BM method, when the output current from the cell returns to the original tank, the amount of salt removed during deionization will be multiplied by the total volume of the tank ($V_{s}$) in the concentration changes according to the following equation.

(27) $${\mathrm{SAC}}_{\mathrm{bm}}=\frac{\mathrm{the}\;\mathrm{amount}\;\mathrm{of}\;\mathrm{salt}\;\mathrm{removed}\left(\mathrm{mg}\right)}{\;\mathrm{electrode}\;\mathrm{mass}\left(g\right)}=\frac{\left(C_{i}-C_{o}\right)\cdot V_{s}}{m_{\mathrm{elc}}}$$

In a deionization process, the charge-discharge cycle can have different times. For example, a cycle with a very short time and a very low salt absorption rate or a very long charging time in which the electrode reaches equilibrium. In this case, the SAC value is known as the maximum salt adsorption capacity (mSAC) or equilibrium adsorption capacity (eqSAC). In a capacitive deionization test to achieve equilibrium of salt absorption, by applying a constant voltage and holding it until the electrode reaches full charge or saturation to absorb salt, no excess ion absorption occurs and the amount of salt concentration in the output of the cell remains constant. When the electrode reaches saturation or equilibrium, the electrical conductivity of the water remains constant. It is important to note that the amount of mSAC depends on the amount of voltage applied, the amount of initial concentration, and the type of salt [75]. Many studies use a voltage of 1.2 volts to apply to the CDI cell, in which case the maximum amount of salt absorption occurs without causing additional reactions such as water electrolysis. The optimum initial salt concentration for adsorption in the CDI process is between 5 and 50 mmol (0.5–5 ms/cm) [144]. Measurements of mSAC have been reported mostly in experiments using sodium chloride (NaCl). However, the mSAC criterion can also be used for other monovalent salts, provided that the type of salt used must be reported because, for example, the molecular weight of KCL salt is 74 mol/g, while sodium chloride salt is equal to 58.4 mol/g which with the same test conditions the amount of salt absorption for KCL is more than NaCl salt [42]. Measuring the mSAC value for waters with different compositions (seawater, etc.) that have different molar masses is very complex and the electrical conductivity of the fluid at the cell output cannot be used, and for this purpose, chromatographic analyses are needed to determine the percentage of adsorption of each ion [78]. In Table 2, the salt adsorption capacity was mentioned under different conditions in CDI.

### 7.2. Average Salt Absorption Rate

In the previous section, the mSAC parameter referred to the amount of salt adsorption by the electrodes but did not provide any information about the salt adsorption rate. Therefore, the second important numerical parameter to describe the function of CDI cells and evaluate the average rate of salt removal from the fed fluid into the cell is introduced as the average salt adsorption rate (ASAR) [27,139]. The ASAR value is obtained by dividing the SAC value by the total charging time (Equation (26)), the value of which is reported in mg/g.min. mg represents the amount of salt absorbed, *g* represents the total mass of the electrode and min represents the total duration of one cycle (charge and discharge) of the capacitive process.

(28) $${\mathrm{ASAR}}_{\mathrm{sp}}=\frac{\mathrm{the}\;\mathrm{amount}\;\mathrm{of}\;\mathrm{salt}\;\mathrm{removed}\left(\mathrm{mg}\right)}{\mathrm{Electrodes}\;\mathrm{mass}\left(g\right)\times \mathrm{charging}\;\mathrm{time}}=\frac{\dot{Q}\cdot \int^{\;}\left[C_{i}-C_{o}\left(t\right)\right]\mathrm{dt}}{m_{\mathrm{elc}}\cdot \left(t_{\mathrm{cf}}-t_{\mathrm{ci}}\right)}$$

**Figure 18:**
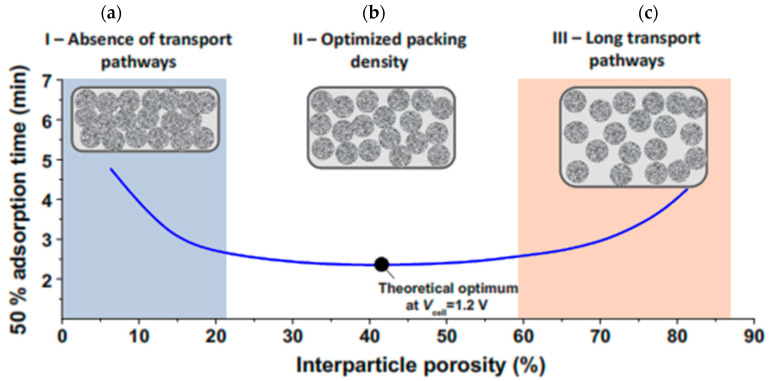
Effect of electrode density and compression on the time required for maximum salt adsorption capacity. (**a**) Compressed and limited state for ion movement, (**b**) Optimal electrode compression state, (**c**) Non-compressed state and maximum porosity space between particles. Reproduced from Ref. [78] with permission from the Royal Society of Chemistry.

Equation (28) show the ASAR value for the conditions in which the system operates in SP mode, where the values $t_{\mathrm{ci}}$ and $t_{\mathrm{cf}}$ indicate the start and end time of the charging phase. The concept of ASAR has been defined by some researchers as the rate of water desalination [22,53]. In the previous section, it was explained that the SAC value depends on the properties of the electrode and is part of the properties of the electrode. Unlike SAC, however, the amount of ASAR depends on many system factors, including charging time, initial fluid concentration, electrode thickness, materials used to make the electrode, and cell structure. Therefore, specifying the exact test conditions when reporting the ASAR value is very important [78,138]. Considering the proper charging time in the deionization cycle when a constant voltage is applied to the cell can have the greatest effect on the ASAR value [27]. When a constant voltage is applied to the cell, the salt concentration of the fed solution gradually decreases to a balanced value. Applying a charge time less than the amount required to reach equilibrium will increase the ASAR. The second factor that affects the amount of ASAR is the salinity of the fed fluid. Increasing the salinity causes the electrodes to charge and saturate rapidly, which in turn increases the amount of ASAR. Before examining the effect of electrode thickness, it is necessary to describe the effect of the materials used to make the electrode. The material used to make the electrode can affect the ASAR value in several ways. When materials with less porosity than microporous porosity (less than nanoparticles) are used, more salt uptake is possible, which increases ASAR [81]. However, the negative effect that the sub-nanoparticle size of the electrode can have on ASAR is that in a very small porous space, the dynamic transfer particles of ions are limited, which increases the charging time of the electrode and, as a result, reduces the amount of ASAR [157]. Electrode thickness also has an adverse effect on ASAR. As the electrode thickness decreases, the ion path can be reduced so it can achieve a better rate of deionization and increase the amount of ASAR [158]. The simultaneous effect of electrode thickness, density, and porosity space between electrode particles can combine to have complex and different effects. The effect of density and compression in the porosity space on reaching the minimum time required for salt adsorption capacity at capacitive cell electrodes is shown in Figure 18 [78]. Depending on the size of the device and the technology of making the electrodes, their thickness varies. However, in general, most CDI tests use an electrode with a thickness of 100 to 500 μm [34]. As can be seen, there is no empty space for the ions to move and the electrode is fully compressed. In this case, the maximum time is needed to reach the salt absorption capacity, so the amount of ASAR is reduced in this case. As the electrode compression decreases and the porous space increases (about 42%), optimal conditions are provided for the electrode, in which there is the minimum time required for the saturation state of the absorption capacity, and thus the ASAR value increases. 

**Figure 19:**
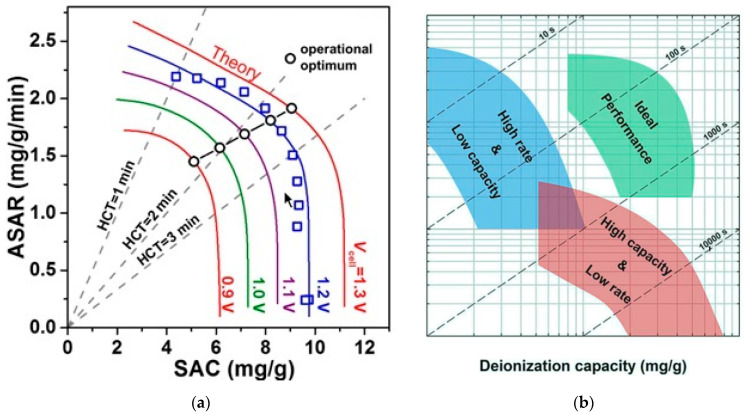
Kim-Yoon diagram for the average rate of adsorbed salt versus salt removal capacity. (**a**) Optimal points of different applied voltages. Reproduced from Ref. [78], with permission from the Royal Society of Chemistry. (**b**) Optimal areas for ASAR and SAC variables. Reproduced from Ref. [143]. with permission from the Royal Society of Chemistry.

The last parameter that can have the greatest impact on the ASAR value is how the electrodes are arranged. As described in Section 4.2, the placement of the electrodes is in two ways: Flow-Through and Flow-By. In the Flow-Through method, the ASAR value will be higher than the Flow-by mode due to the minimization of the transverse distance between the electrodes, which leads to a decrease in the cell resistance and consequently a reduction in the charging time [22]. Inspired by the Ragone chart (a graph showing the size of energy storage versus energy delivery rate) in 2015, Kim and Yoon plotted the average rate of salt absorbed versus salt removal capacity (Figure 19). The ASAR versus SAC chart, also known as the Kim-Yoon chart, integrates the two important indicators for CDI evaluation discussed in the previous section into one chart. In this diagram, the time required in the charge phase and the discharge phase are considered to be the same, and the diagram is based on the required half-cycle-time (HCT) for one cycle. As shown in Figure 19a, the SAC value will be the lowest possible to reach the maximum ASAR value. Therefore, to achieve the optimal state of CDI cell working conditions, using the Kim-Yoon diagram will be very useful and practical. The black circles indicate the optimal points for each applied voltage obtained by multiplying the ASAR value by the SAC [159]. According to the figure, with increasing the amount of voltage applied to the cell, the amount of HCT also increases, which increases the amount of salt absorption. Figure 19b shows the optimal regions for both variables.

### 7.3. Charging Efficiency

The third key parameter in evaluating a CDI system that compares the amount of salt absorbed versus the amount of charge stored on the electrodes is known as the charge efficiency [160]. The concept and importance of the ratio between the amount of salt removed and the amount of charge stored was first proposed by Johnson and Newman in 1971 to describe the exact electrical absorption behavior of ions with the shape of an EDL [37]. The term charge efficiency firstly was used by Avraham et al. in 2009 [83,161]. Zhao et al. in 2010, used Λ to indicate the amount of charge efficiency [130], and the title and symbol have been used ever since [37,83,130,161]. Charging efficiency is a critical parameter in evaluating the amount of electrical energy used to remove the number of ions in the CDI system and fixed type electrodes. The charge efficiency is expressed as the ratio between the number of ions that are electrically absorbed by the electrode to the amount of charge that is transmitted in the electrical circuit [162]. Ideally, the charge efficiency should be one, but in practice, this does not happen because the electric charge on the electrodes is able to absorb ions of the same name from the solution and repel ions of the same name from the EDL, which reduces the SAC value and consequently the charge efficiency [163]. To calculate the charge efficiency, the amount of charge transferred is divided by the Faraday constant (F = 96,485 C/mol) according to Coulomb [89]. For a CDI system with the SP transfer method, the charge efficiency value is obtained from the following equation [132].

(29) $$\Lambda_{\mathrm{sp}}=\frac{F\cdot \dot{Q}\cdot \int^{\;}\left[C_{i}-C_{o}\left(t\right)\right]\mathrm{dt}}{\int^{\;}\mathrm{Idt}}$$

In this equation, the integral mentioned in the denominator is equal to the total charge transferred during the deionization process. The following equation is used to obtain the amount of charge efficiency in a system by the BM transfer method [75].

(30) $$\Lambda_{bm=}\frac{F\cdot V_{s}\cdot \left(C_{i}-C_{o}\right)}{\int^{\;}\mathrm{Idt}}$$

The amount of charge efficiency depends on the amount of cell voltage at the time of charge and discharge and the amount of salt concentration of the fed solution. As the amount of charge voltage increases, the charge efficiency increases [159]. Charging efficiency is inversely related to the amount of salt concentration. In general, this parameter is clearly used to compare the energy required by the system.

### 7.4. Current Efficiency

In some cases, CDI systems use current efficiency instead of voltage to be able to more accurately show the amount of energy to remove salt. This parameter is used in electrodialysis systems [88,164]. Current efficiency refers to the rate of salt uptake relative to the flow through the CDI system. Current efficiency is used when the variables of the CDI system, such as the flow and the amount of output concentration, are constant and unchanged, as is the case with the SP method for water deionization [78]. Similar to the voltage efficiency equation, current efficiency is inversely related to the amount of electrical energy used to remove salt. The value of current efficiency for SP systems is obtained from the following equation:

(31) $$\lambda_{\mathrm{sp}}=\frac{F\cdot \dot{Q}\cdot \left(C_{i}-C_{o}\right)}{I}$$

The values of current efficiency as well as charge efficiency can be used as useful and practical feedback to improve CDI tests and optimize the deionization unit.

Coulombic efficiency is another indicator used in CDI systems that shows the ratio of output charge during the cell discharge phase to the amount of input charge to the cell, which is usually less than one, which means that the Faraday reaction can occur in the CDI system [78].

Another indicator that may be used in a CDI system or any water desalination system is defined as the water recovery ratio, which is the ratio of the volume of deionized water to the total volume of water fed.

## 8. Materials Used in Fabricated CDI Cell Electrodes

Everything that has been discussed so far has all been about the absorption of salt from water and its quantity and quality. How to absorb and separate salt from the solution was described in detail in the previous sections. The most important part and structure of a capacitive system is the place where the ions are placed to separate from the water. Undoubtedly, the electrode and its material play the most important role in the applicability and operation of a CDI cell. With the advent of the CDI method from the 1960s until today, the development of this technology is in two parts: scientific concepts—theory and discussion of materials used in the fabrication of electrodes, and the 30-year break in this technology was due to the lack of quality materials. Since 1995, the most work has been carried out on materials to make electrodes, and all research is underway to identify the material that can have the best properties. The most important properties that a material must have to make an electrode in the deionization industry in order to expect the best performance from the system are as follows:

1. It has a high specific surface area in order to be able to absorb ions better, which leads to increasing the adsorption capacity of salt ions [99].
2. Ability of fast movement of ion particles inside the porous space that can absorb many ions without restricting the kinetic movement of ions [165].
3. High electrical conductivity to reduce the contact resistance between the electrode and the collector to prevent voltage drop and energy loss and also to charge the entire contact surface of the electrode without increasing the voltage [119,166].
4. High chemical stability in different voltage and pH ranges for system stability and long electrode life [167].
5. High hydrophilicity behavior to ensure the utilization of the total porosity in the deionization process [117].
6. Low cost in high production capacities [168,169].
7. Flexibility and formability to make electrodes with different shapes [12].
8. Not harmful to the environment with the availability of a large amount of raw material [34].
9. High mechanical resistance in contact with the base conductor plate as well as in contact with the fluid passing through the cell [117,167].
10. Low tendency to scaling and fouling.

Based on the characteristics described above, having high specific surface properties and the possibility of moving particles at the same time seems very complicated because having a high specific surface area requires a large amount of porous space within a tiny size, which limits the movement of particles. On the other hand, a large porous space for fast particle motion reduces the specific surface area. In addition to the surface area, the porosity size, the porosity volume, the connections between the porous spaces, and the proper distribution of the porosities all directly affect the performance of a capacitive system and are of great importance [75,132]. An ideal ion absorption requires a high specific surface area with the size of the pores so that the ions can move easily [34]. The International Union of Pure and Applied Chemistry (IUPAC) divides the size of porosity into three categories based on size [7,170].

1. Macropores with a size larger than 50 nm
2. Mesopores with a size between 2 to 50 nm
3. Micropores less than 2 nanometers in size, also known as nanopores [171].

**Figure 20:**
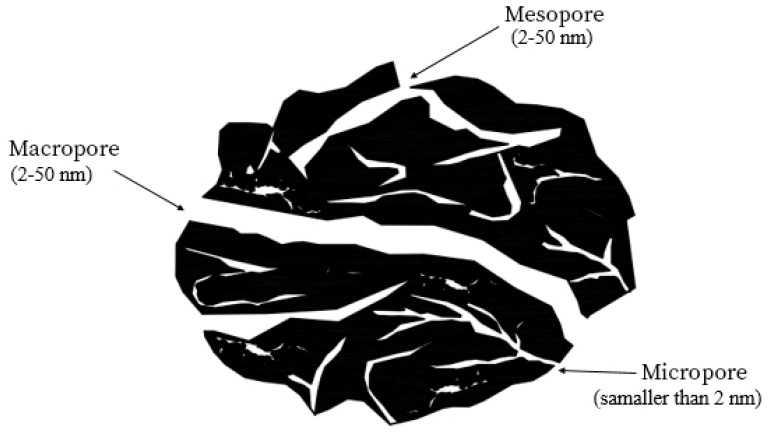
The structure of porous space and its naming types.

Using the average size to indicate porosity is not very common because the porosity of the material is distributed over a wide range, and the use of micro and macro terms in addition to showing the porosity size is also used to indicate the shape of the porosity [82,172]. As shown in Figure 20, macropores are channels that allow ion particles to move between smaller, soluble porosities. Micropores are also very small pores in the skeleton of porous materials where the ions of soluble particles are firmly located. The porosity structure of the material as well as the size distribution of the porosity is considered as an important parameter for the construction of the electrode in the development of capacitive method in the process of deionization of water [173].

**Figure 21:**
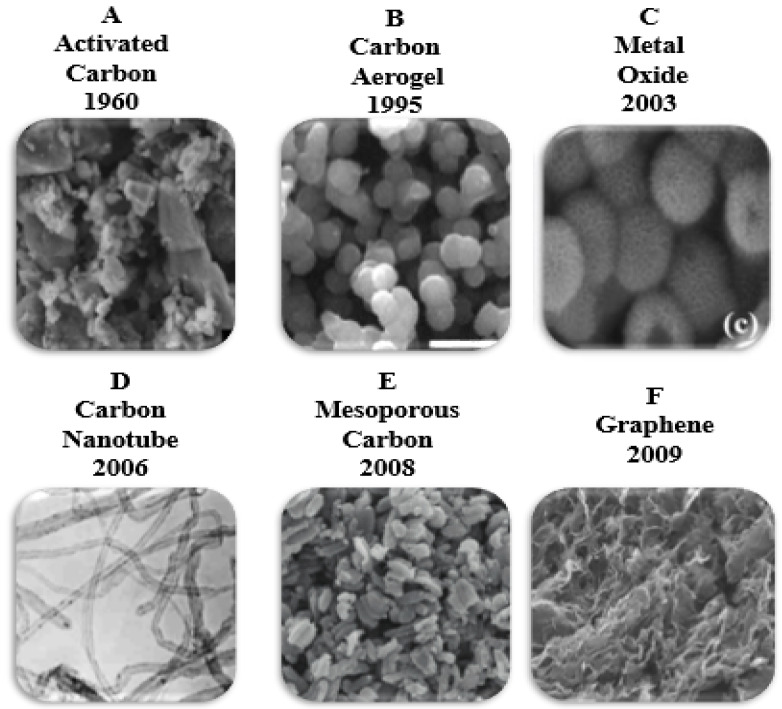
Development process and materials used to make the electrode in the capacitive process: (**A**) Activated carbon, Reprinted/adapted with permission from Ref. [117]. 2013, Luciano et al. (**B**) Carbon Aerogel, Reprinted/adapted with permission from Ref. [176]. 2006, Li et al. (**C**) Metal oxides, Reprinted/adapted with permission from Ref. [177]. 2013, Bian et al. (**D**) Carbon Nanotubes, Reprinted/adapted with permission from [178]. 2006, Zhang et al. (**E**) Mesopores carbon, Reprinted/adapted with permission from Ref. [179]. 2019, Liu et al. (**F**) Graphene, Reprinted/adapted with permission from Ref. [173]. 2020, Han et al.

According to the specifications mentioned above and in theory, electrodes that can be used for the deionization process should have high porosity and high conductivity. However, the choice of materials for the electrode is typically limited by cost, porosity, high surface area, and availability. However, to have the most deionization performance, choosing the correct electrode fabrication material is very key and important [174]. So far, carbon-based materials, metal oxides, and conductive polymers have been widely used to make electrodes in the CDI process [119,175]. Also, in some cases, to improve the characteristics of the electrode, combining these materials and making nanocomposite electrodes has been used. Figure 21 shows the progress of most of the materials used to make CDI electrodes.

Among the materials mentioned, carbon-based materials were used in the early 1990s to make electrodes for energy storage devices such as supercapacitors. These materials had acceptable properties and their function was based on the storage of ions in the EDL and showed that ions can be separated from the solution by this method and mechanism. Therefore, the use of these materials in the CDI method was highly considered. These materials generally showed acceptable performance for the electrical absorption of ions. Since then, a lot of research has been conducted on the use of these materials in the deionization process, leading to the introduction of different types of carbon-based materials. Table 3 shows the desalination performance of different active materials for CDI under different operating modes.

### 8.1. Activated Carbon

Among carbon-based materials, activated carbon (AC) has been used extensively in the fabrication of CDI electrodes since the beginning of the development of the capacitive deionization method in the 1990s. According to the characteristics of a suitable material for making electrodes mentioned in the previous chapter, AC with a very high specific surface area (500–3500 m^2^/g) is used as a common material in making electrodes [215]. AC has low cost (50$/kg) because of its abundant resources, which come from high-carbon natural materials such as wood, coconut husk, coal, or the extraction of synthetic materials such as resins or natural raw materials that are converted to charcoal when exposed to atmospheric air [119,216]. Activated carbon has been the most researched for electrode fabrication due to its properties and easy fabrication as well as its use in large-scale applications [165]. The carbon obtained from the mentioned materials is initially in the form of powder the size of a micrometer, which has a great variety in the size and structure of the porosity and the chemical properties of the surface. This carbon powder was made into a thin film and used as the first electrode in the deionization industry. The electrode made of pure AC had a very low capacity and electrical absorption due to the irregular structure of porosity and low conductivity of activated carbon [99,217]. Therefore, many studies on chemical actions and mixing instructions on activated carbon have been performed and are still ongoing in order to improve the properties of higher salt adsorption capacity, structural properties (surface area increase), and hydrophilic behavior. To increase the adsorption capacity of activated carbon, the researchers tried to improve this property by increasing the performance of the CDI process by adding substances such as naphthalene and benzyl alcohol to the activated carbon of granular type [218]. Other researchers by adding potassium hydroxide [17], nitric acid [63], uranium [219], and titanium dioxide [54] increased the adsorption capacity due to increasing the surface area, enlarging the pores, and reducing the Faraday reactions from 5 to 10% for the electrodes made of activated carbon. To improve the electrical absorption property, modifications were made to the activated carbon by increasing the absorption capacity by increasing the charge transfer rate [58]. Other researchers have added changes to nanoparticles to improve electrodes made of activated carbon for capacitive deionization [216,220,221]. These are just a few of the many studies that have been conducted on powdered or fibrous activated carbon. Extensive research has been conducted in this field, which has led to the introduction of various materials that can be referred to for further study, which is beyond the scope of this article.

### 8.2. Carbon Aerogel

Another group of carbon-based materials commonly used in the manufacture of CDI process electrodes is known as carbon aerogel. This type of carbon is made of a porous material with spherical nanoparticles [99]. Aerogel carbon due to significant properties such as high electrical conductivity (10–100 S/cm), very low resistance (400 Ω·m/cm), medium specific surface area (400–1100 m^2^/g), very low density (<0.1 g/mL), unique fabric structure (interconnected particles with a diameter of 3 to 30 nm with in-network porosity) and the amount of porosity in the mesoporous range (<50 nm) as an acceptable material is being used widely in the capacitive deionization process [12,34,52,75,165,222,223]. Aerogel carbon is formed by the chemical process of sol-gel in powder forms, thin films, and small, solid granules. Aerogel carbon was first introduced in 1995 by Farmer et al. For use in the CDI industry, and their research removed 99% of the salt during water desalination [43,44]. Using a solution containing sodium chloride and sodium nitrate, they tested the carbon ion desalination performance at different voltages and reported that the salt absorption capacity decreased by 6 to 8 percent at 1.2 volts after one month of operation. They also reported that the electrical conductivity of aerogel carbon is strongly influenced by pyrolysis process parameters such as time and temperature. One of the remarkable properties of carbon aerogel is that it has a very light weight due to its low density and therefore has a very low mechanical strength. Adding silica to carbon aerogel improves its hydrophilicity and mechanical properties. Also, a 28% increase in the amount of electrical absorption capacity was obtained by adding silica to the aerogel carbon [7]. By synthesizing carbon aerogel integrated with adjustable porosity, its high specific surface area and mechanical strength were increased, and this property was obtained by performing a one-step sol-gel process [224]. In order to improve the performance of the aerogel carbon electrode, some researchers made this type of electrode by electrospinning method, which increased the electrical absorption capacity and also made it easier and faster to prepare the aerogel carbon electrode for use in the CDI process [214,225,226,227,228]. In addition to the positive properties of aerogel carbon for electrode fabrication in the capacitive process, further development and use are very limited due to the difficult preparation process and high cost, low porosity size (which causes overlap in the EDL), and sedimentation of the aerogel surface by natural organic matter (therefore in natural water desalination) [99,119,216].

### 8.3. Graphene

Graphene first attracted the attention of researchers in 2004 with the separation of graphite by ultra-thin peeling as the newest material introduced in materials science [229]. Graphene is a layer of graphite about the thickness of the diameter of an atom. Graphene has a two-dimensional structure in which the atoms are placed together in a flat plate in the form of hexagonal carbon rings that form the honeycomb structure. This thin layer is formed by chemical vapor deposition or exfoliation. Among carbon-based materials, graphene has significant physicochemical properties such as very high specific surface area (2630 m^2^/g), very high electrical conductivity (2000 S/cm), high thermal conductivity, good mechanical stability, and porosity structure. In addition to being considered in the use of electrical systems, energy storage devices, and sensors, it has been studied as one of the most important materials for making CDI electrodes [230,231,232,233,234,235,236,237]. Unlike activated carbon, the surface area available to absorb graphene ions is outside the material and on the surface, while in activated carbon the entire surface area (slit pores) is completely inside the particles [34]. In addition to much attention in industry and academia, this material was very popular in the fields of energy and water desalination [117]. However, due to the structure of nanoparticles of these materials, by combining them and also because graphene sheets can be bent or shrunk, they can have seam porosity even on one of their plates [7]. Also, due to the plate property and single-layer structure, graphene ions can move faster than other carbon materials [99]. The first use of aerogel carbon at the CDI in 2009 was made by Lee et al. by preparing porous graphene with a smooth surface by the hydrazine hydrate reaction method and applying a voltage of 2 volts at which the salt adsorption capacity was 1.85 mg/g [60]. The first study on the electrical absorption performance of an electrode made of graphene in the CDI process showed that the ion removal rate was higher than that of an electrode made of activated carbon [165,238]. However, due to the two-dimensional structure of graphene due to the oxidation-reduction reaction, it causes the accumulation of ions and, consequently, reduces the contact surface, reduces the electrical conductivity, reduces the ion movement rate, and thus reduces the ion adsorption capacity [60,95,239]. Therefore, a lot of research has been conducted to synthesize the three-dimensional structure to prevent this problem and the results have shown that the three-dimensional structure can have a high specific surface area, high porosity volume, high adsorption capacity, and optimal performance compared to the two-dimensional state in a capacitive deionization process and is also acceptable for removing heavy metals and salt ions from wastewater [16,240,241]. Because graphene is a very new material with unique properties, it has received a lot of attention in the field of CDI and over the last ten years, more than seventy articles have been published in this field. To get acquainted with this substance and its use in the CDI industry, Liu et al. in 2017 had an extensive overview of this issue, which can be referred to this article for further study [217].

### 8.4. Ordered Mesoporous Activated Carbon

Another carbon-based material used in the deionization industry is known as Ordered Mesoporous Activated Carbon (OMCs). This type of material is structurally very different from activated carbon. As mentioned earlier, the transfer of ions within the structure of carbonaceous materials has a large effect on the performance of capacitive deionization. Increasing the specific surface area causes very small porosity, which overlaps the electrical double layer and does not seem very desirable for ion absorption and transport. Therefore, electrodes with a mesoporous structure (2 to 50 nm) can have better desalination performance [242,243]. OMCs were first introduced in 2008 as a material in which ions move easily and the deionization process is well performed and has received much attention since then [54]. OMCs are referred to because their structure has a regular porosity that is alternately hexagonal or cubic in shape, which facilitates the transfer of ions within the porous lattice [244]. To make an OMCs electrode, you need templates or molds in which nanoscale carbon particles are placed in that mold. These moldings are carried out in two ways, hard or soft molding, whose basic principles are the same. The raw material carbon is formed inside the samples and then the original sample is extracted by chemical or physical methods [245]. In the hard molding method, a pattern sample such as zeolite or silica is leaked into the carbon material and then carbonized. Then, in the final stage, the sample is removed by chemical method (by hydrofluoric acid) and OMCs are obtained [7,34]. In the soft method, which is also a new method, a spontaneous assembly of three-block copolymers with carbonization produces OMCs [99]. Depending on the conditions that occur in the preparation phase, the surface area of OMCs is between 750 and 1500 square meters per gram [9]. As mentioned, due to the unique porosity structure of these materials in which the movement of ions is easier and these conditions are favorable for the capacitive deionization method. However, due to the high cost of manufacturing these materials and the time-consuming construction, and also because it is not economically viable on a commercial scale, these conditions have led to restrictions on the production of capacitive electrodes with these materials [119]. 

### 8.5. Carbide-Derived Carbons

To improve the performance of carbon-based materials in chemical processes, and because there were some problems such as high electrical resistance, low ion capacity, and weak ion bonding in activated carbon, some modifications in the porosity structure and combining some different gas groups (H_2_, CO_2_) was considered [99,246,247]. Therefore, some material properties such as hydrophilicity, high thermal resistance, surface area increase, and porosity structure of activated carbon were obtained. Carbide-derived carbon (CDCs) are a type of enhanced material that, unlike OMCs, is a very fine-grained, microporous material (0.5 to 2.2 nm) [174]. Unlike OMCs, the porosity of these materials is not intermittent and is very thinly distributed and is easily activated from carbon and other carbon-based materials can be distinguished. CDCs are usually guided by fertilizing carbide powder in dry chlorine gas at temperatures between 200 and 1200 °C, followed by annealing hydrogen gas to remove residual chlorine compounds, after which CDCs are formed [34]. The specific surface area of these materials is in the range of 1200 to 2000 square meters per gram, but this amount can be expanded up to 3000 square meters per gram [47]. Although these materials have sub-nano pores, which increase the specific surface area, as mentioned earlier, these pores restrict the passage of ionic particles.

### 8.6. Nanotube Carbon

Carbon nanotubes (CNTs) are another type of carbon-based material used to fabricate CDI electrodes. These materials were first introduced in 1991 by Lijima and Ichihashi [248]. CNTs are defined as a cylinder of carbon with a wall diameter of nanometers. These unstitched tubes, which are longer than their diameter, are made by tubing a graphene plate and are single-walled carbon nanotubes or multi-walled carbon nanotubes [216]. Single-layer nanotubes are nested together by van der Waals forces to form a multilayer nanotube with a diameter of about 5 to 20 nanometers. CNTs are made from chemical vapor condensation (CVD). In this method, the decomposition of hydrocarbon gases is used along with metal catalysts [178]. Both single-layer and multi-layer CNTs are used to make electrodes in the CDI process and have very high conductivity and maintain good ion absorption capacity during long charge and discharge periods [249,250]. The surface area for CNTs is about 120 to 500 square meters per gram, and some researchers have reported an increase in surface area for carbon nanotubes by adding KOH [251,252]. However, all surface areas of these materials are available for ion adsorption, and this has led to the development of CDI electrodes with these materials. However, due to its hydrophobicity and tendency to accumulate ions, it causes a lack of access to all surfaces, and hence the deionization efficiency decreases. CNTs have a smaller surface area than activated carbon but are more conductive than activated carbon and can have a lower adsorption capacity on their own [253]. On the other hand, activated carbon has low conductivity in the construction of the electrode, so by combining activated carbon and CNTs, a combination of high surface area and high conductivity is obtained [99,194]. Gao et al. proposed a new type of electrode made from a combination of CNTs and nanofiber carbon that is relatively expensive to make and is used to attract a variety of monovalent and polyvalent ions [56]. A new example of CNTs was introduced by Wang et al. in 2011, known as the sponge nanotube carbon. This material is obtained by the chemical vapor condensation process and has a very flexible property, three-dimensional structure with mesoporous porosity, very high conductivity, and high specific surface area. In their study of the deionization process, Wang et al. claim that the deionization capacity of this material was approximately 50% higher than that of other carbon materials used to make electrodes, but that this material is not yet commercially available [254]. In addition to the extensive research that has been conducted to make CNTs as electrodes in CDI, there are limitations, such as the presence of metal catalysts in the structure of CNTs, which reduce chemical stability and thus lack the correct and accurate reaction in ion adsorption [255]. Also, these materials have not yet been widely used because they are not economical in the manufacture of electrodes [238,256].

### 8.7. Carbon Black

Carbon black is obtained from the thermal decomposition or oxidation of hydrocarbons. This material is obtained from the incomplete burning of plants and fossil fuels [214]. More than 80% of this substance is placed in the atmosphere as smoke [257]. This material has a very good powder property and has been highly regarded in many industries. The particle shape of this material is spherical and due to its good mechanical and thermal resistance, it is used as a filler and reinforcement for polymer parts [117]. Due to its very small size (20 to 200 nm) is used as a suitable material to create porosity in nanocomposites [258]. However, the use of this material in the capacitive deionization method is due to its very high conductivity (0.1 to 100 S/cm) and is used in the fabrication of electrodes as an additive to increase the conductivity of the electrode [119,143,259,260,261]. Carbon black’s surface area is in the range of 10 to 1500 square meters per gram [262,263]. The combination of this material with activated carbon can lead to the introduction of electrodes with high specific surface properties and high conductivity, which is very desirable for use in capacitive deionization.

## 9. Conclusions

The perspectives of human life and the advances that have been made in recent decades have multiplied the need for water and energy, and the demand for this has become one of the main daily needs of human beings. The issue of water and energy dependence has long been considered, and the restriction of access to water by energy has been a major factor in not meeting this basic human need in various fields, including agriculture, industry, and the domestic field. Finding a solution that can achieve the water quantity needed for resources without environmental and energy constraints has become very important. The capacitive deionization method, which has been much discussed and researched in the last two decades, can compete with other water desalination methods that have a high consumption for desalination with the least energy consumption. Given that this method is very new and innovative, research and data collection for this topic and help future researchers is very useful. In this article, the latest developments and the main principles of the CDI method are collected in order to be of help to future researchers in this regard.

## Figures and Tables

**Table 1:** The progress of theoretical and conceptual development in CDI systems for water desalination.

Num.	Progress Process (Conceptual or Theory)	Year	Ref.
1	Electrochemical demineralization	1960	[26]
2	Electrochemistry of carbon	1961	[28]
3	The mechanism of demineralization at carbon electrodes	1966	[29]
4	Mathematical theory of electrochemical demineralization	1967	[30]
5	Activated carbon used in desalination	1968	[93]
6	Electrochemically controlled ion exchange	1969	[32]
7	Electrochemically controlled ion exchange	1969	[94]
8	Electrochemical desalination of brackish water	1968	[31]
9	Electric Double Layer theory (EDL)	1970	[35]
10	Porous carbon electrodes	1971	[37]
11	Four-action electrochemical parametric pumping cycles	1978	[39]
12	Carbon aerogel electrodes	1995	[43]
13	Membrane exchange in front of electrodes (MCDI)	2006	[66]
14	Time-dependent ion selectivity in porous electrodes	2012	[67]
15	Science of water desalination by capacitive deionization	2013	[34]
16	Water desalination via capacitive deionization	2015	[78]
17	Carbon-based composite materials	2015	[95]
18	Resistance in Capacitive Deionization	2016	[96]
19	Redox active porous electrodes in CDI	2017	[97]
20	Faradaic reactions in capacitive deionization	2018	[98]
21	Electrode materials for CDI	2020	[99]
22	CDI and RO desalination	2020	[100]
23	Ion intercalation materials in CDI	2020	[101]
24	Faradic capacitive deionization	2021	[102]
25	Flow-electrode capacitive deionization	2022	[103]

**Table 2:** Comparison of the Salt Adsorption Capacity (SAC) reported in the literature.

Type of Operation [Batch or Single Pass]	Initial Concentration of Feed Rate (mg/L)	Flow Rate (mL/min)	Applied Voltage (V)	Salt Adsorption Capacity (mg/g)	Electrode Material	Specific Capacitance/(F/g)	Ref.
Single pass	5844	Na	1.2	30.2	Activated carbon with Anion	200	[145]
Single pass	584	Na	0.7	15.6	Activated carbon	Na	[146]
Batch	3000	30	1.7	Na	Carbon nanotubes	108/1	[147]
Single pass	4000	60	1.2	30	NoritSX Ultra Activated Charcoal	Na	[148]
Batch	1020	40	1.2	3	carbon nanotubes and carbon nanofibers	Na	[79]
Batch	1000	Na	1.4	66.15	Activated carbon	193.694	[149]
Batch	600	10	1.2	28.62	Gr phene/CNTs/ZnO	280	[150]
Batch	1000	50	1.2	14.91	Nitrogen-doped porous carbonspheres	290.74	[151]
Batch	500	25	1.2	15.31	Herein, hollow ZIFs-derived nanoporous carbons	243	[152]
Single pass	584	10	1.2	31.5	sodium manganese oxide	300	[109]
Batch	50	10	1.4	22.15	Tungesten Carbide Graphene Nanoflakes	580	[153]
Batch	500	Na	1.2	21.32	Activated carbon	246.6	[154]
Single pass	4000	22	1.2	13	MXene	132	[155]
Batch	600	10	1.2	17.8	manganese oxide/ fabricated-AC	388	[156]

**Table 3:** Desalination performance of different material electrodes with CDI in the literature.

Electrode Material	Surface Area of Material (m^2^/g)	Specific Capacitance (F/g)	Flow Rate/(mL/min)	Applied Voltage (V)	Initial Concentration (mg/L)	Desalination Capacity (mg/g)	Ref.
activated carbon	576	60.6	-	1.2	292	11.26	[180]
activated carbon	24.3	-		1.8	1000	128.6	[181]
activated carbon	246.7	138.5	-	1.8	1000	167.4	[182]
activated carbon	1968	-	-	1.6	50	4.6	[16]
activated carbon	-	-	-	1.2	198.5	3.5	[84]
activated carbon	-			1.2	292	6.9	[57]
activated carbon	-	169.1	-	1.2	25	0.25	[54]
activated carbon	-	-	25	1.2	35	176.7	[183]
Activated carbon cloth	2794	125		1.2	292	16	[184]
Highly porous activated carbon	2254	309	40	1.2	500	16.3	[185]
Carbon aerogel	460.34	74.23	-	1.2	400	270.59	[186]
Carbon aerogel	-	-	-	1.2	496	2.9	[44]
Carbon aerogel	-	-	-	1.5	3000	9.6	[22]
Carbon aerogel	113	-	-	1.3	2000	7	[53]
Carbon aerogel	910	83	-	1.2	200	5.62	[187]
Carbon aerogel	-	-	25	1.2	500	10.34	[188]
Carbon aerogel- activated carbon composite	1100	90	-	1.2	1000	17	[189]
Aerogel activated CO_2_	1069	100.5	8	1.2	6200	11.8	[190]
Graphene	-	-	-	2	250	8.6	[191]
Graphene	-	-	40	2	3000	1.85	[60]
Graphene	-	-	10	1.2	100	29.5	[192]
Graphene	315.6	65	-	1.2	500	29.18	[193]
Carbon nanotubes	129.368	-	-	1.2	3000	1.734	[194]
graphene/MnO_2_	-	292	-	1.2	70	5.01	[195]
Graphene doped with nitrogen	358.9	253.06	-	1.8	100	4.8	[196]
Carbon nanotubes-graphenehybrid	435	-	-	1.6	4000	79.4	[197]
Nitrogen-Doped Graphene	918	56.2	-	1.4	500	18.4	[198]
Ordered Mesoporous Carbon	1491	192	-	0.8	46	0.93	[199]
Highly ordered mesoporous carbon nano-polyhedra	750	130	-	1	116.8	14.58	[200]
Graphene-Na4Ti_9_O_20_ nanotubes (AC/rGO@NTO)	142.43	120.45	-	1.4	250	41.8	[201]
Graphene nanocomposite	-	-	20	1.2	55	2.5	[202]
Graphene chitosan-Mn_3_ O_4_ (Gr- Cs- Mn_3_ O_4_ composite)	-	190	-	1.6	300	12.7	[203]
Graphene mesoporous carbon	685.2	89.55	25	2	40	0.7	[204]
3D porous graphene	680	70	-	1.4	300	14.32	[205]
Graphene doped with nitrogen	358.9	253.06	-	1.8	100	4.8	[196]
Microporous graphene	3513	20	-	2	74	11.86	[206]
Nanoporous three-Dimensional Graphene	-	200	-	1.6	500	17.1	[207]
reduced graphene- carbon nanotubes aerogel	-	-	-	1.6	4000	79.4	[197]
Resorcinol-based MC-coated graphite	488	-	30	1.2	5000	15.2	[42]
carbon polymer composite	952	168.2	30	1.2	1500	14.2	[208]
Sulfonyl- N-doped porouscarbon	844	215.3	15	1.2	40	15.5	[209]
Hierarchically porous carbon	2185.71	-	25	1.4	40	34.27	[210]
Carbon nanotubes- Si-Ag	77.907	149.1	1	0.8	210	21.5	[211]
hole-rich graphene skeleton	-	219.9	15	2	572	29.6	[212]
three-dimensional channel-structured graphene	711.9	207.4	-	2	295	9.6	[213]
N-hierarchical porous CA	2405	153	25	1.2	500	17.9	[214]
nitrogen-doped porous carbon spheres	1640	290.74	50	1.2	1000	14.91	[151]

## Data Availability

No publicly archived data was established.
